# Discovery and mechanistic insights of dibenzoylmethane as a broad spectrum inhibitor of coronavirus

**DOI:** 10.1371/journal.ppat.1013492

**Published:** 2025-09-08

**Authors:** Yuan Sun, Xiaoyang Shu, Lu Chen, Weijuan Shang, Yumin Zhang, Gengfu Xiao, Leike Zhang

**Affiliations:** 1 State Key Laboratory of Virology and Biosafety, Wuhan Institute of Virology, Chinese Academy of Sciences, Wuhan, China; 2 University of Chinese Academy of Sciences, Beijing, China; 3 Hubei Jiangxia Laboratory, Wuhan, China; Trinity College, IRELAND

## Abstract

Coronavirus, a large family of positive-sense RNA viruses, are responsible for both mild and severe respiratory illnesses, ranging from the common cold to life-threatening conditions. Despite significant advances in vaccine and antiviral development, the high mutability of human coronaviruses (HCoVs), such as SARS-CoV-2, presents a major challenge in treating these infections. Effective, broad-spectrum antiviral drugs are urgently needed to address both current and future HCoV outbreaks. Here, we conducted high-throughput screening of a natural product library containing 3407 compounds to identify potential antiviral agents against HCoV-OC43 and HCoV-229E. We identified several natural products with inhibitory effects on HCoV-229E, HCoV-OC43, and the SARS-CoV-2 variants Delta (B.1.617.2) and Omicron (BA.5) *in vitro* without evident cytotoxicity. Among these, dibenzoylmethane (DBM) not only demonstrated broad-spectrum anticoronavirus activity *in vitro* but also effectively inhibited HCoV-OC43 replication in a BALB/c mouse model. Pharmacokinetic analysis revealed that DBM, when administered orally, maintained effective concentrations in the blood over an extended period, suggesting its suitability for oral administration. Mechanistically, DBM was found to regulate caspase-6, a host factor that suppresses interferon signalling and promotes HCoV replication. These findings highlight DBM as a promising candidate for the development of therapeutics targeting HCoVs, offering potential for treating infections by both established and emerging HCoVs.

## Author summary

In this study, we investigated the potential of dibenzoylmethane (DBM), a natural compound, as a therapeutic agent against human coronaviruses (HCoVs), including HCoV-OC43 and HCoV-229E, as well as more recent SARS-CoV-2 variants. Our findings demonstrate that DBM effectively inhibits viral replication and exhibits broad-spectrum antiviral activity. Through mechanistic analysis, we identified that DBM functions by modulating the host protein caspase-6, which is crucial for viral-induced suppression of the immune response. In animal models, DBM was shown to reduce viral replication without causing significant toxicity, highlighting its therapeutic potential. These results suggest that DBM could serve as a valuable candidate for the development of broad-spectrum antiviral treatments, particularly in response to emerging coronavirus infections. Our study underscores the promising role of natural products in antiviral drug discovery, offering a novel approach to combat viral diseases, especially in the context of rapidly evolving pathogens.

## Introduction

Coronaviruses (CoVs) constitute a large family of single-strand and positive-sense RNA viruses. Historically, several coronaviruses, such as SARS-CoV, MERS-CoV, and SARS-CoV-2, have caused significant public health crises. In addition to these more notorious viruses, other human coronaviruses, including HCoV-OC43 and HCoV-229E, are common pathogens responsible for mild symptoms such as fever, rhinitis, pharyngitis, and abdominal discomfort [1,2]. However, these viruses can also lead to severe acute respiratory infections, such as pneumonia and bronchitis, and may be life-threatening in immunocompromised individuals, including children, adults over 65 years of age, and patients with underlying conditions such as cardiopathy, chronic obstructive pulmonary disease, diabetes, and immunosuppression [3]. Notably, SARS- and MERS-like CoVs found in bat populations have demonstrated the ability to efficiently infect primary human airway cells [4,5], underscoring the potential for future cross-species transmission of CoVs.

Currently, effective treatments for coronavirus infections are limited, particularly given the high variability of these viruses. The efficacy of existing antiviral drugs and vaccines may be diminished or even rendered ineffective as the virus undergoes mutation [6]. For example, during the COVID-19 pandemic, SARS-CoV-2 generated multiple variants that exhibited significant differences in transmissibility, pathogenicity, vaccine escape, and drug resistance [7,8]. In this context, the development of broad-spectrum antiviral drugs is highly important.

One common approach for discovering antiviral compounds is high-throughput screening (HTS) of compound libraries [9]. In contrast to the process of de novo drug development, drug repurposing presents numerous advantages, including a reduced timeline for development, sustained safety profiles, a lower incidence of adverse effects, and increased cost-effectiveness. These attributes render drug repurposing a promising strategy for discovering therapeutic options for both emerging and re-emerging infectious diseases. Natural products (NPs) encompass a vast array of biologically active compounds. Some NPs have been proven to be ideal reservoirs for repurposing against viral infection [10–13]. However, most of these NPs have shown mild to modest effects, and very few of them have been investigated or found to be effective *in vivo* with favourable pharmacokinetics and treatment routes.

In this study, we carried out high-throughput repurposing screening of a natural product library containing 3407 compounds to identify active antivirals against HCoV-OC43 and HCoV-229E. In total, 5 NPs inhibited both OC43 and 229E *in vitro* without obvious cytotoxicity. Among these compounds, DBM showed broad-spectrum anti-HCoV activity *in vitro* and potently suppressed HCoV-OC43 replication in BALB/c mice. Moreover, the pharmacokinetic and metabolic stability studies suggested that DBM is a safe and well-absorbed compound for oral treatment. Mechanistically, we found that DBM exerts anticoronavirus activity by regulating caspase-6, a host factor that suppresses interferon (IFN) signalling and facilitates coronavirus replication. Taken together, our results suggest that DBM is a potential candidate for combating HCoV infection.

## Results

### Identification of hits through HTS that prevent cell death caused by coronavirus infections

**Fig 1:**
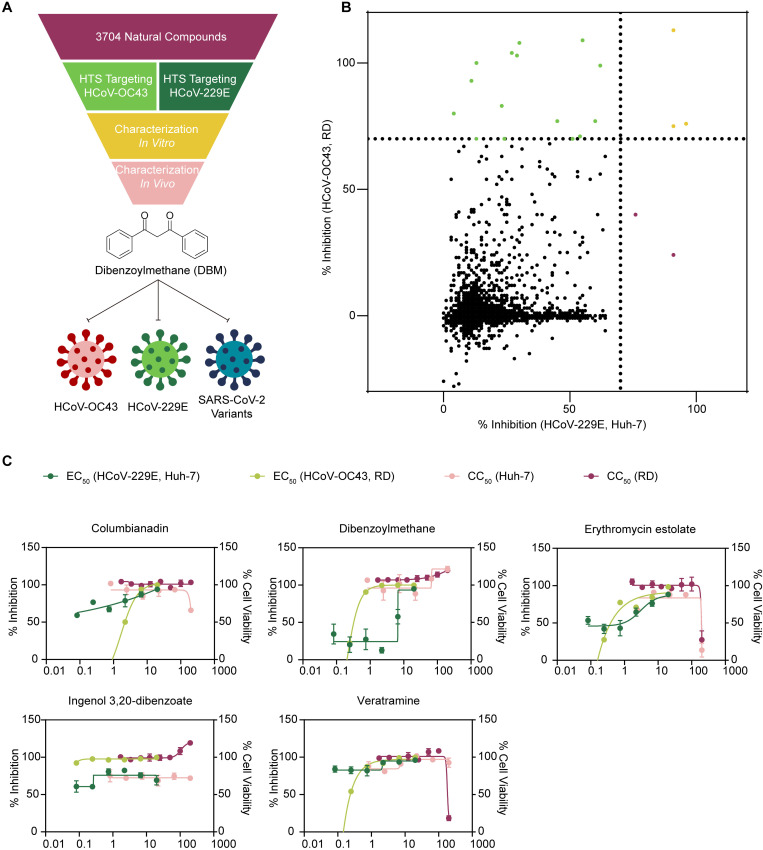
High-throughput screening of antiviral natural compounds. **A)** Flowchart outlining the high-throughput screening process. The flowchart was created by the authors using Adobe Illustrator 2022. **B)** Inhibition of HCoV-229E and HCoV-OC43 by compounds from the natural product library. Each dot on the scatter plot represents an individual compound. Compounds exhibiting inhibition rates greater than 70% at 10 μM are highlighted. **C)** Half-maximal effective concentration (EC_50_) values for HCoV-229E and HCoV-OC43, and 50% cytotoxic concentration (CC_50_) values of the natural compounds in Huh-7 and RD cells. To calculate the EC_50_, cells were co-incubated with a gradient dilution of the compounds 1 h prior to inoculation with the corresponding viral strains. After 24 h of infection, viral RNA copies in the supernatants were analysed using qRT-PCR. CC_50_ values were tested as described in materials and methods. The experiments were repeated three times independently with similar results.

HTS has become an indispensable tool in modern drug discovery, enabling the rapid evaluation of large compound libraries for potential therapeutic activity. By systematically testing thousands of compounds, HTS provides a powerful means to identify candidates with specific biological effects, including antiviral properties. In this study, we developed an HTS platform to evaluate the effectiveness of compounds that protect cells from cell death caused by two common human coronaviruses, HCoV-229E and HCoV-OC43. We screened a library of 3407 compounds from the Natural Product Library Plus (MedChemExpress, HY-L021P) with the CellCounting-Lite 2.0 Reagent (Vazyme, DD1101) to assess cell viability after inoculation with the viruses in the presence or absence of natural compounds from the library (Fig 1A). Through this screening, 20 natural compounds were identified to be able to protect cells during infection with HCoV-229E and/or HCoV-OC43 (Fig 1B). We subsequently determined the 50% effective concentration (EC_50_) values of the 20 valid compounds for inhibiting HCoV-229E in Huh-7 cells and HCoV-OC43 in RD cells by analysing the amount of viral RNA released by the infected cells. Furthermore, the 50% cytotoxic concentration (CC_50_) was assessed with the Cell Counting Kit-8 (CCK-8, GlpBio), and compounds with high selectivity indices (SIs), including columbianadin, DBM, erythromycin estolate, ingenol 3,20-dibenzoate, and veratramine, were selected for further evaluation (Fig 1C). These compounds exhibited antiviral effects on both HCoV-229E and HCoV-OC43, suggesting their potential broad-spectrum anticoronavirus activity. Compounds such as (2S)-2’-methoxykurarinone, (R)-sulforaphane, amphotericin B, baohuoside I, cyclovirobuxine D, deserpidine, euphorbia factor L7a, levistolide A, neoglycyrol, ponicidin, sophoraflavanone G, and tetrandrine exhibited high cytotoxicity in this assay, resulting in poor SIs below 25 (S1A Fig). Consequently, these compounds were not further assessed, as it is challenging to develop compounds with low SIs into viable drugs.

### *In vitro* identification of the anticoronavirus activity and cytotoxicity of the five selected compounds

**Fig 2:**
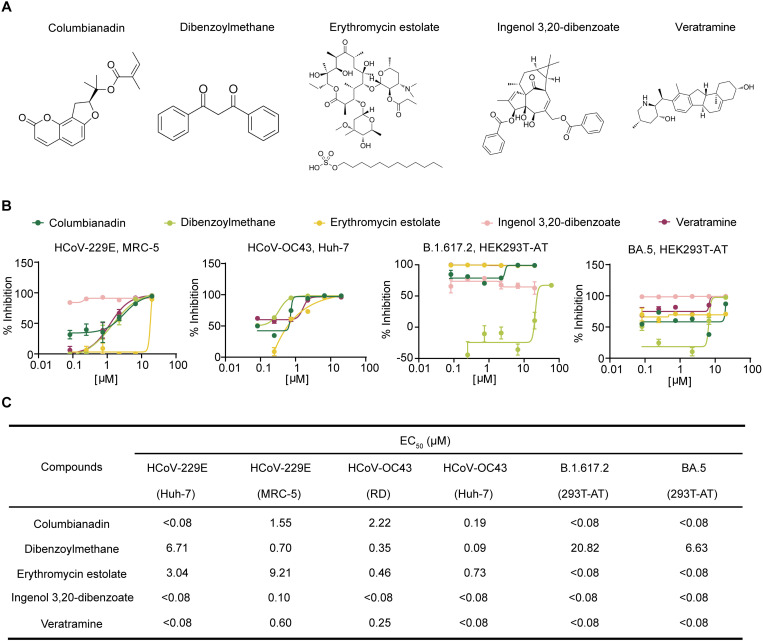
Columbianidin, DBM, erythromycin estolate, ingenol 3,20-dibenzoate, and veratramine exhibit anti-coronavirus effects *in vitro.* **A)** Chemical structures of the five compounds. **B)** Antiviral efficacy against HCoV-229E, HCoV-OC43, and SARS-CoV-2 variants B.1.617.2 (Delta) and BA.5 (Omicron) of the five compounds. **C)** Table of EC_50_ values for the five compounds inhibiting HCoVs across various cell lines. The experiments were repeated three times independently with similar results.

We subsequently validated the inhibitory effects of columbianidin, DBM, erythromycin estolate, ingenol 3,20-dibenzoate, and veratramine against HCoV-229E, HCoV-OC43, and the SARS-CoV-2 variants Delta (B.1.617.2) and Omicron (BA.5). The structural formulas of these compounds are shown in Fig 2A. The EC_50_ values were calculated via qRT-PCR from the number of viral RNA copies released into the culture medium 24 hours postinfection in the presence or absence of the test compounds. The antiviral efficacy and cytotoxicity of the five compounds against each virus strain were examined in at least two different cell lines (Fig 2B and 2C). All tested compounds effectively inhibited replication of viruses HCoV-229E, HCoV-OC43, and the SARS-CoV-2 variants Delta (B.1.617.2) and Omicron (BA.5) in cell lines. Moreover, DBM was found to effectively inhibit another alpha coronavirus HCoV-NL63 with an EC_50_ value of 11.66 μM (S1B Fig). The CC_50_ values of the five compounds were assessed in the MRC-5, HEK293T-ACE2-TMPRSS2 (HEK293T-AT), and Vero E6 cell lines (S1C and S1D Fig).

### Screening of natural compounds in a suckling mouse model of HCoV-OC43 infection

**Fig 3:**
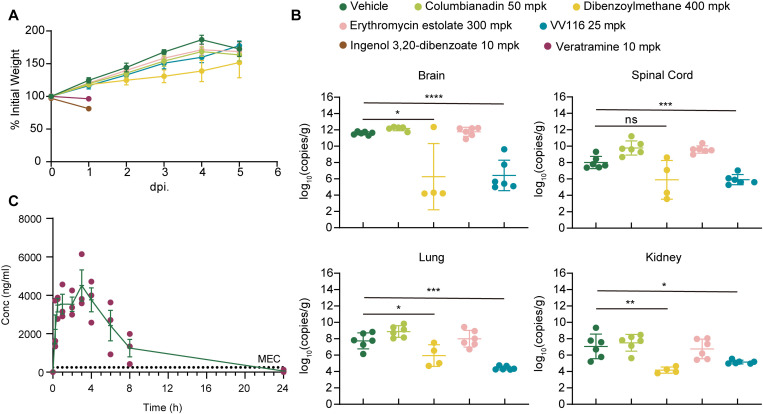
Characterisation of the antiviral effects of columbianidin, DBM, erythromycin estolate, veratramine, and ingenol 3,20-dibenzoate in animal models. **A)** Body weight changes in HCoV-OC43-infected BALB/c suckling mice. Mice in each group were inoculated with HCoV-OC43 1 h before the first drug administration and body weight data were recorded daily. **B)** Viral copy numbers detected in homogenised organs and tissues collected from infected mice on Day 5. After grinding and centrifuging the samples with DMEM, viral copies from the supernatants were tested by qRT-PCR. **C)** Concentration-time curve of rats administered 200 mg/kg of DBM orally. Plasma samples of rats were collected for analysing the concentration of DBM in them 0 min, 15 min, 30 min, 1.0 h, 2.0 h, 3.0 h, 4.0 h, 6.0 h, 8.0 h, and 24.0 h after the administration. DBM concentrations in plasma were determined using LC-MS/MS. Data were statistically analysed with Student’s T test. *: p ≤ 0.05, **: p ≤ 0.01, ***: p ≤ 0.001, ****: p ≤ 0.0001; ns, not significant. The experiments were repeated three times independently with similar results.

After confirming the antiviral activity of the five selected natural compounds *in vitro*, we further assessed their therapeutic efficacy in a suckling mouse model of HCoV-OC43 infection. In this experiment, suckling mice were randomly divided into seven groups and treated with different agents. Columbianadin has previously been reported to suppress airway inflammation in mice at a dose of 50 mpk [14]. Feeding 1% DBM in the diet was found to be safe for mice in antitumour studies [15]. Doses of erythromycin estolate exceeding 300 mpk and of veratramine exceeding 10 mpk have also been safely used in antitumour mouse models [16,17]. VV116, which has been shown to exhibit good anticoronavirus activity at a dose of 25 mpk [18], was used as the positive control compound in this experiment. A group of mice receiving menstruum orally quaque die (QD) was used as the control group. The other groups were treated with columbianadin at 50 mg/kg (mpk) QD, DBM at 400 mpk QD, erythromycin estolate at 300 mpk QD, veratramine at 10 mpk QD, ingenol 3,20-dibenzoate at 10 mpk QD, or VV116 at 25 mpk QD. Mice were inoculated with 1 × 10^4^ TCID_50_ of HCoV-OC43 on the first day of the experiment and then orally treated with either a placebo or the test compounds on the first and following days. The groups of veratramine- and ingenol 3,20-dibenzoate-treated mice were euthanized on Day 1 because veratramine and ingenol 3,20-dibenzoate exhibited toxicity in the suckling mice. All the mice were sacrificed on Day 5, and the brains, spinal cords, lungs, and kidneys were collected from the mice. HCoV-OC43 infection resulted in significant body weight loss on Day 5, whereas mice from the DBM- and VV116-treated groups did not show any body weight loss during the experiment (Fig 3A). The number of viral copies from collected mouse organs and tissues was analysed by qRT-PCR to detect the HCoV-OC43 nucleocapsid gene sequence. Compared with the vehicle group, the DBM group presented significantly fewer viral copy numbers in the brain, lungs, and kidneys at 5 days post infection (Fig 3B). The viral load in the brains of the DBM-treated group was approximately 6 logs lower than that in the vehicle group. Moreover, treatment with columbianidin and erythromycin estolate did not significantly reduce the number of viral RNA copies in HCoV-OC43-infected mice.

Among the five natural compounds, only DBM had a protective effect on HCoV-OC43-infected mice. Thus, a pharmacokinetic study was performed for DBM in Sprague-Dawley (SD) rats. Following oral administration of menstruum or DBM at 200 mpk, blood samples from the rats were collected at 2 min, 5 min, 15 min, 30 min, 1 h, 2 h, 4 h, 8 h, and 24 h posttreatment. The concentration of DBM in the plasma was determined by liquid chromatography with tandem mass spectrometry (LC-MS/MS). The minimum effective concentration (MEC) line in the graph represents the EC_95_ value of DBM (Fig 3C). During the test, DBM reached its Cmax of 4,760 ± 1,920 ng/mL at 2.33 ± 1.15 h, with an AUClast of 34,300 ± 14,800 ng h/mL and a T_1/2_ of 2.91 h (S2A Fig). To analyse the metabolic stability of DBM, we incubated human and mouse liver microsomes with DBM. The T_1/2_ of metabolism was an average of 13.92 min in human liver microsomes and 4.84 min in mouse liver microsomes. The results revealed that DBM was more stable when in human liver microsomes than in mouse liver microsomes (S2B Fig).

### *In vivo* characterization of the anti-HCoV-OC43 activity of DBM

**Fig 4:**
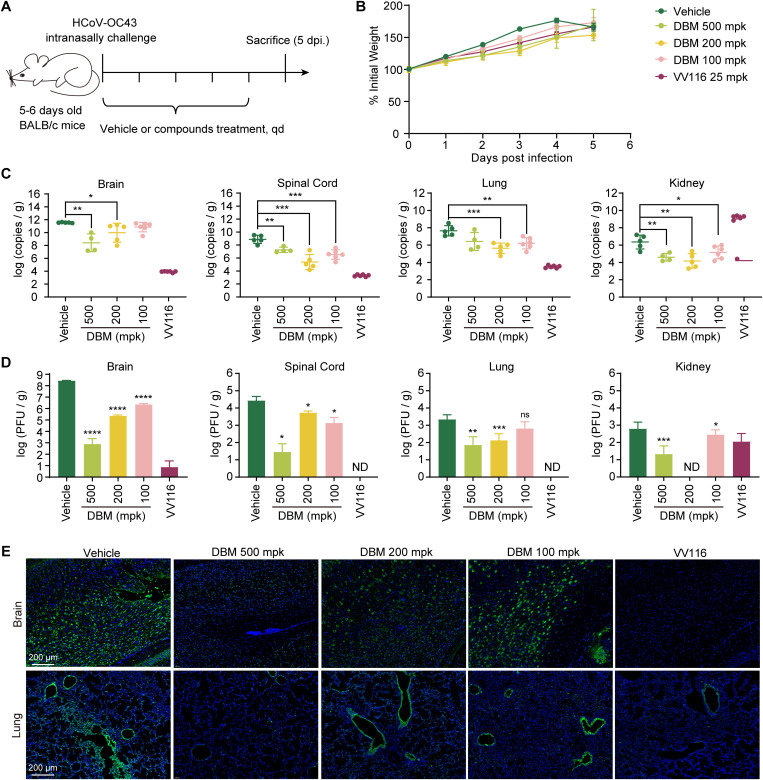
*In vivo* antiviral efficacy of DBM in 5-day-old BALB/c suckling mice infected with HCoV-OC43. **A)** Schematic of the experimental design for evaluating DBM in suckling mice. Each group consisted of 5-6 suckling mice. The schematic was created by the authors using Adobe Illustrator 2022. **B)** Body weight changes of mice during the experiment. Body weights were recorded daily and all mice were euthanised on the fifth day after infection. **C)** Viral RNA copies in the brains, spinal cords, lungs, and kidneys of mice collected on Day 5. After grinding and centrifuging the samples with DMEM, viral copies from the supernatants were tested by qRT-PCR. **D)** Viral titres in homogenised organs and tissues collected from mice on Day 5. **E)** Immunofluorescence staining of brain and lung sections from mice treated with different regimens. HCoV-OC43 nucleocapsid proteins were detected by immunofluorescence (green). Cell nuclei were stained with DAPI (blue). Data were statistically analyzed with Student’s T test. *: p ≤ 0.05, **: p ≤ 0.01, ***: p ≤ 0.001, ****: p ≤ 0.0001; ns, not significant. The experiments were repeated three times independently with similar results.

Next, we characterized the therapeutic efficacy of the DBM *in vivo*. In this experiment, randomized 5–6-day-old suckling BALB/c mice were orally treated with DBM at concentrations of 500, 200, and 100 mpk 2 hours after being intranasally challenged with HCoV-OC43. Menstruum and VV116 were used as negative and positive controls, respectively. All the mice were sacrificed on Day 5 for organ and sample collection (Fig 4A). The body weight changes of the mice were continuously monitored during the trial. Infection with HCoV-OC43 led to body weight loss in the vehicle-treated mice on Day 5 (Fig 4B). Moreover, the mice treated with DBM or VV116 did not experience weight loss during the experiment, indicating that DBM protected the mice from disease-related symptoms. The number of viral RNA copies from the brain, spinal cord, lungs, and kidneys were analysed via qRT-PCR, which targeted the HCoV-OC43 nucleocapsid gene sequence. On Day 5, the number of viral RNA copies in the vehicle-treated group reached approximately 10^12^ copies in the brain, 10^9^ copies in the spinal cord, 10^8^ copies in the lungs, and 10^6^ copies in the kidney (Fig 4C). In the mice treated with DBM at 500 mpk, the number of viral RNA copies within these organs and tissues was reduced by 1–3 logs compared with the vehicle-treated group. DBM treatment at different concentrations from 100 to 500 mpk resulted in a significant reduction in viral RNA copies in certain organs or tissues. The viral titres of the organs and tissues characterized by the immunoplaque assay also indicated that the administration of DBM at 500 mpk led to a reduction in viral loads (Fig 4D). Sections of mouse brains, spinal cords, lungs, and kidneys were subjected to immunofluorescence staining with antibodies targeting HCoV-OC43 nucleocapsid proteins. The results revealed that DBM treatment reduced the viral loads in certain organs and tissues of HCoV-OC43-infected mice (Figs 4E and S3A).

The cytokine storm caused by severe HCoV-OC43 infection can lead to death in mice. By analysing the gene expression of cytokines (*CCL2*, *CXCL10*, *IL-1β*, *IL-6*, and *TNF-α*), we found that the inflammation accompanied by HCoV-OC43 infection was severe in the central nervous system, including the brain and spinal cord. DBM administration dramatically reduced inflammation in the brain and spinal cord of infected mice (S4A Fig). We subsequently histologically examined the pathological changes in brain and lung sections from infected mice. The results revealed that the oral administration of DBM significantly reduced the damage caused by viral infection in these organs. Compared with vehicle treatment, DBM treatment reduced the accumulation of immune effector cells (black arrows) and severe lesions (red arrows) in the brain and pulmonary fibrosis (black arrows), pulmonary oedema (red arrows) and the formation of sputum (green arrows) in the lungs (S4B Fig).

### DBM inhibits HCoV replication by downregulating caspase-6

To obtain further insight into the stage at which DBM inhibits the replication of HCoV-OC43 and other HCoVs, RD cells were incubated with compounds, including DBM, remdesivir (RDV), and chloroquine (CQ), at different stages of viral infection. Viral RNA from the supernatant was extracted and quantitatively analysed via qRT-PCR. The results revealed that the addition of the DBM at 2 hours after virus inoculation potently inhibited the replication of HCoV-OC43, whereas the incubation of DBM with cells before and during virus infection did not significantly inhibit HCoV-OC43 infection (S5A Fig). For the viral binding assay, cells were cultured in the presence or absence of DBM at different concentrations for 1 hour before being infected with HCoV-OC43 at an MOI of 50, and then were placed on ice for 2 hours for the viral particles to bind to the membrane of the host cells, followed by washing with PBS. The detection of viral RNA from viral particles bound to cells confirmed that the administration of DBM did not interfere with the process of viral binding (S5B Fig). In addition to binding to receptors from host cells, the spike proteins of HCoV-OC43 could also trigger membrane fusion when HCoV-OC43 spike plasmid-transfected 293T cells were seeded together with RD cells as target cells, and the addition of DBM had no effect on this process (S5C Fig). To examine whether DBM inhibits viral internalisation, cells were inoculated with HCoV-OC43 for 1 hour on ice before being treated with either DBM or DMSO, and CQ was included to prevent endosomal acidification. Subsequently, HCoV-OC43 nucleocapsid proteins were immunostained with CoraLite Plus 488 to assess viral internalisation via confocal microscopy. Analysis of the number of viral particles entering the cells indicated that DBM did not interfere with the internalisation process (S5D and S5E Fig). To determine whether DBM influences viral release, we quantified viral RNA both intracellularly and in the supernatant. No significant difference was observed in the proportion of viral RNAs released into the supernatant between DBM-treated samples and controls (S5F Fig), suggesting that while DBM does not impede the release of the virus into the culture medium.

**Fig 5:**
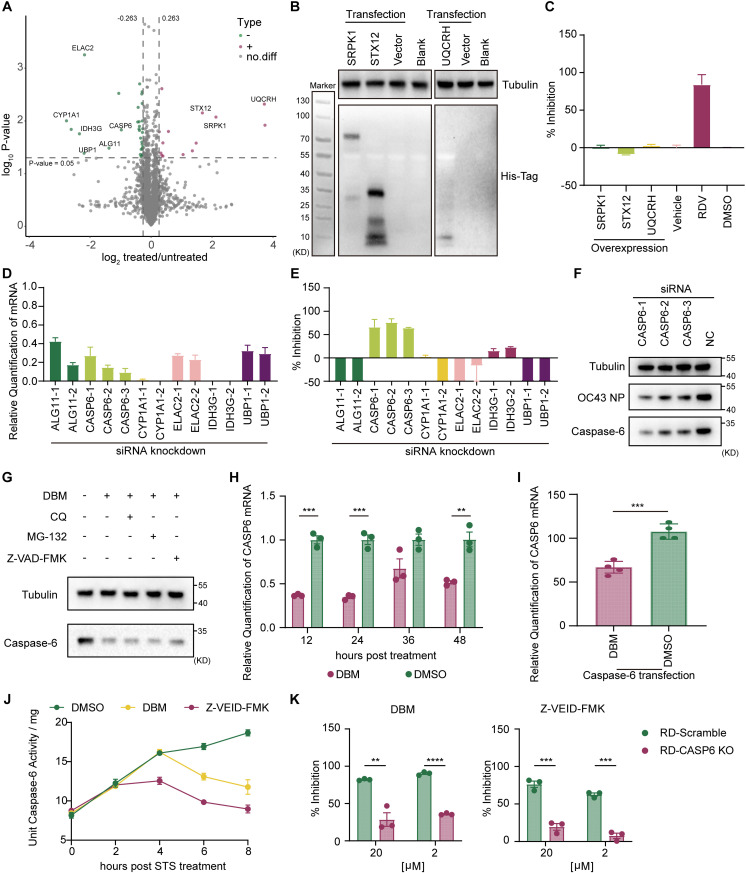
Downregulation of caspase-6 induced by DBM treatment participates in inhibiting HCoV-OC43 replication. **A)** Proteins upregulated and downregulated by DBM treatment. Protein samples were detected by LC-MS/MS after extraction and enzymatic steps, and the measured data were retrieved and analysed by MaxQuant (V1.6.6). Each point in the volcano plot represents a protein whose colour indicates whether the protein was screened as significantly different, with red representing up-regulation and green representing down-regulation. **B)** Protein expression of SPRK1, STX12, and UQCRH in HEK-293T cells transfected with corresponding plasmids. **C)** Antiviral efficacy of the transient expression of SPRK1, STX12, and UQCRH. The corresponding protein expression plasmids were transfected into RD cells by transient transfection 24 h before HCoV-OC43 inoculation. **D)** Relative mRNA content following siRNA knockdown. The corresponding siRNAs were transfected into RD cells separately by Lipofectamine RNAiMAX, and the total RNA in the cells was extracted 24 h after transfection. **E)** Antiviral efficacy of knockdown of DBM downregulated proteins. HCoV-OC43 was inoculated to the mRNA knockdown cells, and the viral RNA in the supernatant was analysed after 24 h of inoculation. **F)** Antiviral efficacy of caspase-6 (CASP6) knockdown, as characterised by Western blotting. Three different siRNAs were used to knockdown the expression of CASP6, respectively, and HCoV-OC43 was inoculated into the cells 24 h after siRNA transfection, and samples were collected 24 h after viral inoculation. **G)** Quantification of caspase-6 protein levels in DBM-treated cells, in the presence or absence of protein degradation inhibitors. Cells were pretreated with 50 μM DBM for 4 h, followed by the addition of CQ, MG-132 or Z-VAD-FMK to the cell culture medium, respectively. DMSO was used as a control. **H)** Quantification of CASP6 mRNA in DBM-treated cells. Cells was harvested at 12h, 24h, 36h and 48h after DBM or DMSO being added to the culture medium. **I)** Quantification of CASP6 mRNA in DBM-treated cells following CASP6 overexpression. Cells were pretreated with DBM or DMSO for 1 h, followed by transfection of Caspase-6 protein expression plasmid into cells by transient transfection, and cell samples were harvested at 24 h after transfection. **J)** Measurement of caspase-6 catalytic activity in cells treated with DMSO, DBM or Z-VEID-FMK, respectively. One unit of enzyme activity defined as the amount of enzyme that can shear 1 nmol of specific polypeptide-pNA to produce 1 nmol of pNA of Caspase in one hour at 37°C when the substrate is saturated. **K)** Viral inhibition efficiency of DBM and Z-VEID-FMK in *CASP6* knockout cells pool. Data were statistically analysed with Student’s T test. *: p ≤ 0.05, **: p ≤ 0.01, ***: p ≤ 0.001, ****: p ≤ 0.0001; ns, not significant. The experiments were repeated three times independently with similar results.

To identify the intrinsic mechanism of the distinct antiviral phenotypes of DBM, quantitative proteomics studies were conducted via LC-MS/MS to identify the proteins whose expression was upregulated or downregulated by DBM treatment (Fig 5A). Serine/arginine-rich splicing factor protein kinase-1 (SRPK1), syntaxin 12 (STX12) and ubiquinol-cytochrome c reductase hinge protein (UQCRH) from the upregulated group have been reported to be related to the infection and replication of certain kinds of viruses [19–21]. Nevertheless, the overexpression of these three proteins did not significantly inhibit HCoV-OC43 infection (Fig 5B and 5C). Proteins downregulated by DBM treatment, including isocitrate dehydrogenase (NAD (+)) 3 noncatalytic subunit gamma (IDH3G), ALG11 alpha-1,2-mannosyltransferase (ALG11), caspase-6 (CASP6), cytochrome P450 family 1 subfamily A member 1 (CYP1A1), ElaC ribonuclease Z 2 (ELAC2), and upstream binding protein 1 (UBP1), were knocked down in host cells by siRNA transfection, resulting in a significant reduction in the relative mRNA levels of each gene compared to the control group 24 hours post-transfection (Fig 5D). The replication efficiency of HCoV-OC43 in siRNA-transfected cells was evaluated via qRT-PCR (Fig 5E). Three different siRNAs for CASP6 were used for knockdown validation to eliminate the interference of siRNA knockdown efficiency, and the results revealed that successful knockdown of CASP6 significantly inhibited HCoV-OC43 replication (Fig 5F). siRNA-mediated transfection of CASP6–1, CASP6–2 and CASP6–3 before HCoV-OC43 infection reduced the quantity of CASP6 and the HCoV-OC43 nucleocapsid protein in HCoV-OC43-infected cells, indicating that a decrease in caspase-6 in host cells can inhibit virus replication.

To explore how caspase-6 was downregulated by DBM, we blocked the main protein degradation pathway in cells, including the ubiquitin-proteasome system, the lysosomal proteolysis pathway, and the apoptotic pathway, which leads to the cleavage of caspase, with the inhibitors CQ, MG-132, and Z-VAD-FMK, respectively. The Western blot results indicated that DBM-induced caspase-6 downregulation was not related to any of the aforementioned protein degradation pathways (Fig 5G). Moreover, we observed that in DBM-treated cells, the relative level of CASP6 mRNA was decreased starting at 12 hours after compound inoculation, which explained the terminal reduction in the level of the caspase-6 protein (Fig 5H). Interestingly, for cells transfected with the caspase-6 expression plasmid with alternative promoter elements, the relative level of caspase-6 mRNA was also decreased by DBM treatment, which suggested that DBM may affect the mRNA stability of caspase-6 (Fig 5I). Eventually, the decrease in the amount of caspase-6 mRNA and protein led to the decrease in the activity of caspase-6 in DBM-treated cells (Fig 5J). To further validate the important role of caspase-6 in DBM-mediated antiviral mechanisms, we constructed a *CASP6* knockout RD cell line to evaluate the antiviral activity of DBM and Z-VEID-FMK, a peptidomimetic inhibitor that covalently binds to caspase-6. The antiviral activity of both DBM and Z-VEID-FMK was decreased in the *CASP6* knockout cell pools, indicating DBM inhibits viral replication through down-regulation of caspase-6 (Fig 5K). Caspase-6, caspase-3 and caspase-7 are apoptosis-executing caspase, and we found that knockdown of CASP3 or CASP7 expression did not effectively inhibiting HCoV-OC43 replication (S6A and S6B Fig). In addition, (S)-(+)-5-[1-(2-methoxymethylpyrrolidinyl)sulfonyl]isatin (caspase-3/7 inhibitor I), a potent and reversible inhibitor of caspase-3 and caspase-7, was not effective in suppressing viral replication (S6C Fig).

A key advantage of host-targeting antivirals (HTAs) over direct-acting agents (DAAs) is their lower susceptibility to drug resistance. In this study, we serially passaged HCoV-OC43 for 17 generations in the presence of DBM, gradually increasing the DBM concentration from 0 to 4.5 times its EC_50_ value. Next, we evaluated the EC_50_ values of virus strains passaged with or without DBM. The results revealed no apparent differences in the EC_50_ values between the DBM-treated group (0.85 μM) and the DMSO control group (0.57 μM) (S6A and S6B Fig). In summary, caspase-6 was demonstrated to be an important intracellular host factor that could be modulated by DBM to exert its antiviral effects.

### DBM alleviates the cleavage of nucleocapsid proteins to inhibit coronavirus replication

**Fig 6:**
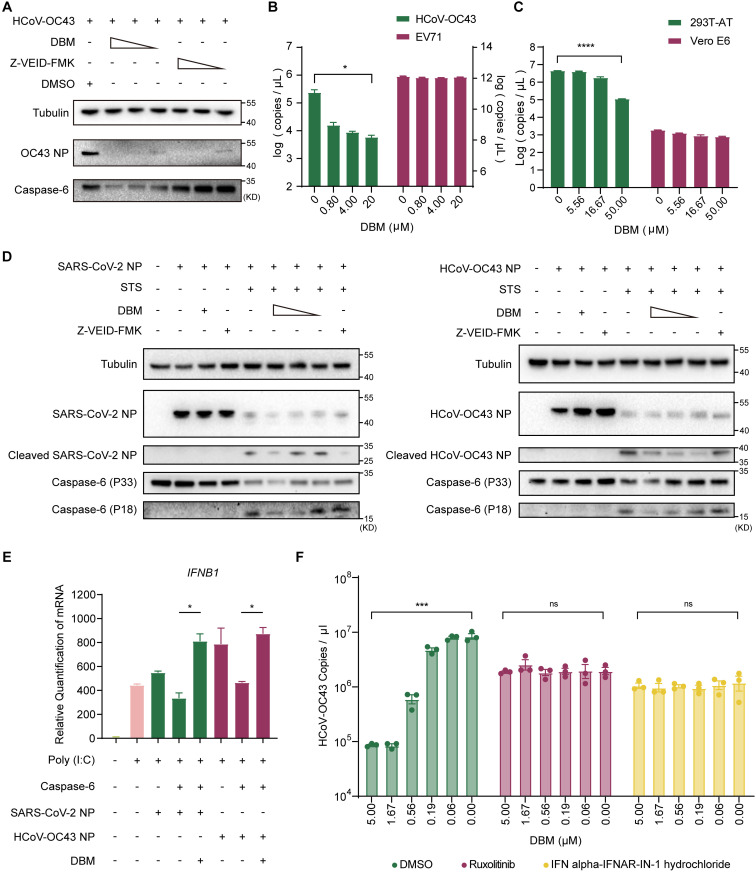
DBM-mediated downregulation of caspase-6 restores IFN signalling suppressed by the coronavirus nucleocapsid protein. **A)** Intracellular levels of HCoV-OC43 nucleocapsid proteins and caspase-6 in HCoV-OC43-infected cells treated with serially diluted DBM or Z-VEID-FMK. Cells were pretreated with gradient-diluted DBM or Z-VEID-FMK prior to viral infection. **B)** Inhibitory effects of DBM against HCoV-OC43 and EV71. Cells were pre-treated with gradient-diluted DBM for 1 hour before inoculated with HCoV-OC43 or EV71, respectively. **C)** Attenuation of antiviral effects of DBM in IFN signalling-deficient cell lines. HEK293T-AT and Vero E6 cells were pre-treated with gradient-diluted DBM for 1 hour before infection. **D)** DBM downregulated caspase-6 and inhibited caspase-6-mediated cleavage of HCoV-OC43 and SARS-CoV-2 nucleocapsid proteins. Cells were pretreated with gradient-diluted DBM or Z-VEID-FMK for 24 h, and then transfected with coronaviruses nucleocapsid proteins expression plasmid, and after 16 h of transfection, cells were treated with DMSO or STS. **E)** DBM alleviated the suppression of *IFNB1* expression induced by coronaviruses nucleocapsid proteins and caspase-6. Cells were pretreated with DMSO or DBM for 24 h, subsequently, transfected with Poly(I:C) and plasmids. **F)** Impact of IFN signalling suppressants on the antiviral activity of DBM. Cells were pretreated with DMSO, Ruxolitinib or IFN alpha-IFNAR-IN-1 hydrochloride, respectively, for 1 hour. Subsequently, a gradient dilution of DBM was added to the medium, and then HCoV-OC43 was inoculated into the cells. Data were statistically analysed with Student’s T test. *: p ≤ 0.05, **: p ≤ 0.01, ***: p ≤ 0.001, ****: p ≤ 0.0001; ns, not significant. The experiments were repeated three times independently with similar results.

To further explore the correlation between the DBM-induced downregulation of caspase-6 and the antiviral effect of DBM, we pretreated cells with serially diluted DBM or Z-VEID-FMK, an effective irreversible caspase-6 inhibitor that causes no reduction in caspase-6 quantity, before HCoV-OC43 inoculation. After infection, the cells were cultured overnight, and the quantities of viral nucleocapsid protein and caspase-6 were analysed by Western blot. The results indicated that both DBM and Z-VEID-FMK inhibited virus replication and that both activity inhibition and downregulation of caspase-6 inhibited coronavirus replication (Fig 6A). Although DBM demonstrated broad-spectrum antiviral effects against a wide range of coronaviruses, namely, HCoV-229E, HCoV-OC43, HCoV-NL63, and the SARS-CoV-2 variants Delta (B.1.617.2) and Omicron (BA.5), DBM exhibited no antiviral activity against other viruses, such as enterovirus EV71 (Fig 6B), which indicated that the antiviral mechanism of DBM may be related to certain unique mechanisms of coronavirus infection. When evaluating the inhibitory effect of DBM against infection with the SARS-CoV-2 BA.5 variant, the antiviral activity was found to be cell line-dependent. DBM demonstrated efficacy in HEK293T-AT cells, but not in Vero E6 cells (Fig 6C). Vero E6 cells, which are commonly used in virological studies, are known to be deficient in IFN production. By assessing the expression of *IFNB1* in SARS-CoV-2 BA.5-infected HEK293T-AT and Vero E6 cells, we observed a marked upregulation of *IFNB1* expression in HEK293T-AT cells, whereas no significant change was detected in Vero E6 cells (S8A Fig). These findings suggest that the antiviral activity of DBM is closely associated with the presence of functional innate immune signalling pathways.

Previous studies have demonstrated that caspase-6 cleaves coronavirus nucleocapsid proteins, generating fragments that act as IFN antagonists and facilitate viral replication [22]. On the basis of this finding, we hypothesized that DBM downregulates caspase-6, reduces the cleavage of nucleocapsid proteins, and thereby enhances IFN signalling to inhibit viral replication. To test this hypothesis, we incubated cells transfected with plasmids expressing coronavirus nucleocapsid proteins in the presence or absence of DBM or Z-VEID-FMK. To simulate the apoptotic environment typically found in coronavirus-infected cells [23], staurosporine (STS), an apoptosis inducer known to activate caspase activity, was added to the culture medium. In the STS-treated groups, apoptosis was successfully induced, resulting in partial cleavage of caspase-6 (zymogen form, P33) to its active form (P18). In the DBM-treated groups, the levels of caspase-6 (P18) were significantly reduced, leading to a decrease in the cleavage of coronavirus nucleocapsid proteins. A similar reduction in cleaved nucleocapsid proteins was observed in the Z-VEID-FMK-treated groups, attributable to the inhibition of caspase-6 activity by Z-VEID-FMK. These results demonstrate that, compared to the control, DBM treatment downregulates caspase-6 and significantly reduces the levels of cleaved HCoV-OC43 and SARS-CoV-2 nucleocapsid proteins (Fig 6D). As the reduction in cleaved nucleocapsid protein fragments could theoretically suppress IFN signalling, we sought to investigate the relationship between DBM treatment and IFN signalling. To this end, we further transfected cells with plasmids expressing both caspase-6 and coronavirus nucleocapsid proteins, and subsequently incubated the cells with or without DBM. Analysis of *IFNB1* and representative IFN-stimulated genes (ISGs), such as *IFIT1*, *IFITM3*, *OAS1*, and *TRIM22*, revealed that DBM reversed the suppression of IFN signalling by caspase-6 and nucleocapsid proteins (Figs 6E and S8B). Whereas the other two apoptosis-executing caspases, caspase-3 and caspase-7, could not inhibit IFN signalling in a similar system (S8C Fig). Furthermore, we examined the expression of *IFNB1* and its downstream IFN-stimulated genes following DBM treatment in the context of HCoV-OC43 infection (S8D and S8E Fig). The results demonstrated that DBM treatment enhanced IFN signalling in HCoV-OC43-infected cells, leading to a significant upregulation of mRNA expression of *IFNB1* and associated ISGs. Additionally, the antiviral activity of DBM was markedly reduced when the cells were pretreated with ruxolitinib (a JAK1/2 inhibitor) or IFN alpha-IFNAR-IN-1 hydrochloride (an IFN-I and IFNAR inhibitor) (Fig 6F) [24,25]. These findings suggested that DBM inhibited coronavirus infection by downregulating caspase-6, thereby reducing the generation of cleaved nucleocapsid protein fragments, which are known to suppress IFN signalling (S9A Fig).

## Discussion

Coronavirus infections have had profound impacts on public health worldwide. However, there are very few broad-spectrum anti-HCoV drugs and vaccines available for clinical use. The COVID-19 pandemic led to an unprecedented international research effort on essentially all aspects of SARS-CoV-2 biology, with high-efficacy vaccines and antivirals produced in record time. The worldwide efforts to combat SARS-CoV-2 have also accelerated studies of pancoronavirus inhibitors due to concerns about known HCoV infections and unknown zoonotic coronaviruses that may be transmitted to humans in the future. While most anti-SARS-CoV-2 agents, such as remdesivir, molnupiravir, and nirmatrelvir, can also inhibit other HCoVs, including OC43 and 229E, all of these approved inhibitors are DAAs. DAAs, while highly specific and potent, face significant limitations. They are prone to resistance due to viral mutations, have a narrow spectrum of activity, and may cause side effects in certain populations. In contrast, by targeting host cellular mechanisms, HTAs reduce the risk of resistance and can provide broad-spectrum antiviral activity. Thus, there is an urgent need for the development of new, broad-spectrum HTAs that target various sites in currently circulating and future emerging HCoVs to prepare for future outbreaks of yet unknown HCoVs.

Natural products are an enormous family of bioactive compounds derived from plants, animals, and microbes, and the most well-known pharmaceutical development of NPs is for anticancer or antiparasitic purposes, such as paclitaxel, ivermectin, and artemisinin [26]. In comparison, the contribution of NPs to the development of antivirals is less prominent. In this study, we conducted an HTS assay to simultaneously identify inhibitors of both HCoV-OC43 and HCoV-229E from a natural product library. Through two rounds of cell-based screening, columbianidin, DBM, erythromycin estolate, veratramine, and ingenol-3,20-dibenzoate were highlighted due to their low cytotoxicity and high inhibitory activity *in vitro*. In suckling BALB/c mice, veratramine and ingenol 3,20-dibenzoate were highly toxic at doses as low as 10 mpk, possibly because of the neurotoxic effects of veratramine and the proinflammatory effects of ingenol-3,20-dibenzoate [27,28]. Our preliminary *in vivo* study indicated that, among the natural products tested, DBM was the only compound that effectively inhibited HCoV-OC43 replication in mice. Further animal studies revealed that oral treatment with 100–500 mg/kg DBM significantly suppressed OC43 viral loads in a dose-dependent manner. Additionally, DBM attenuated inflammation in the central nervous system caused by viral infection. Furthermore, DBM treatment protected brain cells and reduced lung damage in HCoV-OC43-infected mice. Notably, a pharmacokinetic study in SD rats revealed high values for Cmax (4760 ng/ml) and T_1/2_ (2.91 h) when DBM was administered orally, suggesting that DBM could represent a promising candidate for further exploration in antiviral drug development. This study is the first to demonstrate that DBM has anti-HCoV activity, in particular, a potent inhibitory effect against OC43 *in vivo* with favourable pharmacokinetics.

DBM and its derivatives exhibit remarkable potential as multifunctional therapeutic agents, with preclinical evidence highlighting their efficacy in cancer prevention and treatment across diverse models. Their biological activities are underscored by potent cytotoxic effects against cancer cells, achieved through induction of both intrinsic and extrinsic apoptotic pathways. For instance, the derivative DPBP demonstrates selective killing of melanoma cells (IC₅₀ = 6.25 μg/mL) with a high selectivity index, while intercalating into DNA to disrupt its structure [29]. In vivo, dietary DBM profoundly inhibits mammary tumorigenesis in mice, reducing tumor incidence by 97% via suppression of cell proliferation and DNA adduct formation, and similarly arrests the cell cycle in prostate cancer cells at the G₁/S phase [30]. Beyond oncology, DBM acts as a phase 2 enzyme inducer to promote carcinogen detoxification and exhibits antimutagenic properties by inhibiting DNA adduct formation. Notably, preclinical data support its safety, with no signs of hepatotoxicity or nephrotoxicity observed in treated animals [29]. These findings collectively position DBM derivatives as promising candidates for chemoprevention and therapy, particularly for skin, breast, and prostate cancers, though clinical translation requires further investigation into optimal formulations and long-term safety profiles.

The results of a series of cell-based assays proved that DBM inhibits HCoV-OC43 at the postentry and prebudding stages. LC-MS revealed that the expression levels of several host proteins were affected by DBM treatment. Some of the upregulated proteins, including SPRK1, STX12, and UQCRH, have been reported to be relevant to viral infection and replication [19–21]. The downregulated proteins were validated by siRNA knockdown. The reduction in caspase-6 expression inhibited viral replication in HEK293T cells, suggesting that caspase-6 is one of the host factors for OC43 propagation. The downregulation of caspase-6 was proven to be achieved at the mRNA level, as DBM treatment may decrease the quantity of CASP6 mRNA by impairing its stability. On the basis of this evidence, we believe that caspase-6 is the host factor downregulated by DBM during the antiviral process. The virus did not develop significant resistance after 17 consecutive passages in the presence of DBM. This finding suggested that DBM, as an HTA, is not susceptible to drug resistance.

Caspase-6 has been identified as an important host factor for influenza A virus (IAV), as caspase-6 deficiency increases susceptibility to IAV infection both *in vitro* and *in vivo*, as reported previously [31]. However, in the case of coronaviruses, caspase-6 facilitates virus replication by inhibiting the host IFN signalling pathway [22]. The inhibition of caspase-6, whether by decreasing the quantity or by inhibiting enzyme activity, can restrict coronavirus replication. In this study, we found that the downregulation of caspase-6 by DBM further decreased the amount of cleaved coronavirus nucleocapsid proteins, which were reported to be associated with IFN signalling suppression. After DBM treatment, the levels of intracellular *IFNB1* and downstream IFN-stimulated genes were elevated, which could explain why DBM does not function well in IFN signalling-deficient cells or in the presence of IFN signalling suppressants. Given that coronaviruses possess a distinct mechanism for inhibiting IFN signalling, caspase-6 inhibitors have been shown to exhibit antiviral activity against a range of coronaviruses, including SARS-CoV, MERS-CoV, SARS-CoV-2, HCoV-229E, and HCoV-OC43 [22]. Therefore, in addition to the antiviral activity against HCoV-229E, HCoV-OC43, HCoV-NL63, and the SARS-CoV-2 variants Delta (B.1.617.2) and Omicron (BA.5) demonstrated in this study, we hypothesise that DBM may also possess broad-spectrum antiviral effects against other coronaviruses. Moreover, caspase-6 has been implicated in a range of non-viral diseases, with dysregulated caspase-6 activity linked to conditions such as non-alcoholic steatohepatitis, Alzheimer’s disease, Huntington’s disease, and preeclampsia [32–35]. In this study, DBM was identified as a potent inhibitor of caspase-6, suggesting its potential therapeutic application could be extended to the treatment of these diseases.

In summary, we identified a series of active compounds from a natural product library through HTS that effectively inhibited HCoV-OC43 or HCoV-229E. Among them, DBM was demonstrated to broadly inhibit HCoVs, including OC43, 229E, and SARS-CoV-2 variants, *in vitro*. Furthermore, we found that oral treatment with DBM potently inhibited HCoV-OC43 in suckling mice, with the desired pharmacokinetics confirmed in SD rats. Mechanistic studies revealed that the DBM inhibits HCoV-OC43 infection by downregulating the expression of caspase-6, a host factor that facilitates coronavirus replication through the inhibition of the IFN signalling pathway, and that DBM also prevents the cleavage of viral nucleocapsid proteins to suppress the expression of *IFNB1* and IFN-stimulated genes. Our findings demonstrate that DBM is a promising HTA candidate for combating coronavirus.

## Materials and methods

### Ethics statement

All animal experiments were conducted in strict accordance with the guidelines for the use and care of laboratory animals. The experimental protocol was approved by the Ethics Committee of the Wuhan Institute of Virology, Chinese Academy of Sciences (approval no. WIVA25202203).

### Cell lines, viruses and compounds

Huh-7, RD, HEK293T, HEK293T-AT, and Vero E6 cells were maintained in Dulbecco’s modified Eagle’s medium (DMEM, Gibco) supplemented with 10% foetal bovine serum (FBS). MRC-5 cells were cultured in minimum essential medium (MEM) supplemented with 10% FBS. The cells were cultured at 37 °C in a 5% CO₂ incubator and routinely checked for mycoplasma contamination. The viral strains HCoV-229E (ATCC VR-740) and HCoV-OC43 (ATCC VR-1588) were provided by Wuhan University. The SARS-CoV-2 Delta variant (B.1.617.2, IVCAS6.7585) and Omicron variant (BA. 5, IVCAS6.8981) were sourced from the National Virus Resource Center. HCoV-229E, HCoV-OC43, and SARS-CoV-2 variants were propagated in MRC-5, RD, and Vero E6 cells, respectively. All experiments involving authentic SARS-CoV-2 viruses were conducted in the Biosafety Level 3 facility (BSL-3) at the Wuhan Institute of Virology, Chinese Academy of Sciences (CAS).

### HTS of the NP library

High-throughput screening of natural antiviral compounds was conducted with a luminescent cell viability assay. Host cells were plated in 96-well black/clear bottom plates 24 hours prior to the addition of the test compounds at a final concentration of 10 μM. After a 1-hour incubation, the viruses were added to the cell culture medium. At 24 hours postinfection, 100 μL of Cell Counting-Lite 2.0 Reagent (Vazyme, DD1101) was added to each well of the 96-well plates at room temperature. Following a 15-minute incubation, the luciferase activities of each sample were measured with a microplate reader. The inhibition rate was calculated on the basis of the luciferase activity.

### qRT–PCR

RNA extraction was conducted with an automatic nucleic acid extraction instrument (Vazyme, VNP-32P) with a virus DNA/RNA extraction kit 3.0 (Vazyme, RM501–01). The purified RNA samples were subsequently reverse transcribed to cDNA with the PrimeScript RT Reagent Kit with gDNA Eraser (Takara, RR047B), after which quantitative real-time polymerase chain reactions (qRT-PCRs) were performed with TB Green Premix Ex Taq II (Takara, RR820A). The primer pairs used in the qRT-PCR tests were as follows: HCoV-229E NP, F: 5’- CAGTCAAATGGGCTGATGCA -3’ and R: 5’- AAAGGGCTATAAAGAGAATAAGGTATTCT -3’; HCoV-OC43 NP, F: 5’- CGATGAGGCTATTCCGACTAGGT -3’ and R: 5’- CCTTCCTGAGCCTTCAATATAGTAACC -3’; Delta variant RBD, F: 5’- CAATGGTTTAACAGGCACAGG -3’ and R: 5’- CTCAAGTGTCTGTGGATCACG -3’; Omicron variant RBD. F: 5’- CAATGGTTTAAAAGGCACAGG -3’ and R: 5’- CTCAAGTGTCTGTGGATCACG -3’; mouse GAPDH, F: 5’- TGGTGAAGGTCGGTGTGAAC -3’ and R: 5’- GAAGGGGTCGTTGATGGCAA -3’; mouse CCL2, F: 5’-GTGGGGCGTTAACTGCATCT -3’ and R: 5’-GGTCTGAGTGGGACTCAAGG -3’; mouse CXCL10, F: 5’- GGTCTGAGTGGGACTCAAGG -3’ and R: 5’- GTGGCAATGATCTCAACACG -3’; mouse IL1B, F: 5’- TTGACGGACCCCAAAAGATG -3’ and R: 5’- AGAAGGTGCTCATGTCCTCA -3’; mouse IL6, F: 5’- GTTCTCTGGGAAATCGTGGA -3’ and R: 5’- TGTACTCCAGGTAGCTATGG -3’; mouse TNF-α, F: 5’- ATCGGTCCCCAAAGGGATGA -3’ and R: 5’- GCTCCTCCACTTGGTGGTTT -3’; human GAPDH, F: 5’- GAAGATGGTGATGGGATTTC -3’ and R: 5’- GAAGGTGAAGGTCGGAGTC -3’; human ALG11, F: 5’- CATCCATACTGCAATGCTGGTGG -3’ and R: 5’- GACCGTTGACATTAACATCGCCG -3’; human CASP6, F: 5’- AGGTGGATGCAGCCTCCGTTTA -3’ and R: 5’- ATGAGCCGTTCACAGTTTCCCG -3’; human ALG11, F: 5’- CATCCATACTGCAATGCTGGTGG -3’ and R: 5’- GACCGTTGACATTAACATCGCCG -3’; human CYP1A1, F: 5’- GATTGAGCACTGTCAGGAGAAGC -3’ and R: 5’- ATGAGGCTCCAGGAGATAGCAG -3’; human ELAC2, F: 5’- CCAGCATCTGTGCTTGTGGACA -3’ and R: 5’- CTGCGAAGGTTGTGAACTGAGG -3’; human IDH3G, F: 5’- CCAGTGGACTTTGAAGAGGTGC -3’ and R: 5’- CCAGTGGACTTTGAAGAGGTGC -3’; and human UBP1, F: 5’- TCGCTTTGCCAGAGAATCACCG -3’ and R: 5’- GCCGTCCTATGATACCACAATCC -3’.

### Determination of antiviral activity *in vitro*

Huh-7, RD, MRC-5, HEK293T-AT, and Vero E6 cells were initially seeded onto 48-well plates at a concentration of 60000 cells/well and allowed to grow overnight. One hour prior to virus inoculation, the culture medium was replaced with fresh culture medium containing compounds that had been serially diluted. The viruses were then introduced into the culture medium at the optimal multiplicity of infection (MOI). At 24 h after infection, the inoculum was collected to determine the viral RNA copy number via qRT-PCR. The efficacy of the compounds in inhibiting viral replication was assessed on the basis of the viral copy number, and the 50% effective concentration (EC50) was calculated with GraphPad Prism software 8.0. Experiments involving authentic SARS-CoV-2 viruses were conducted at the Biosafety Level 3 facility (BSL-3) at the Wuhan Institute of Virology, Chinese Academy of Sciences (CAS), whereas experiments involving HCoV-229E and HCoV-OC43 were carried out at the Biosafety Level 2 facility (BSL-2) at the same institute.

### Cytotoxicity assay

The cells were seeded into 96-well plates at a density of 40000 cells/well and subsequently cultured overnight at 37 °C in a 5% CO₂ incubator. The compounds were serially diluted before their introduction into the cell culture medium and were incubated with the cells for 24 hours. After the addition of 10 μL of Cell Counting Kit-8 (CCK-8, GlpBio), the supernatant from each well was replaced with 100 μL of fresh culture medium. After the plate was incubated for 2 hours, the absorbance at 450 nm was measured with a microplate reader. The 50% cytotoxic concentration (CC50) was calculated with GraphPad Prism software 8.0 on the basis of the absorbance measurements.

### *In vivo* efficacy of DBM against HCoV-OC43 in suckling mice

BALB/c mice were maintained and bred in a specific-pathogen-free (SPF) environment at the Laboratory Animal Center of Wuhan Institute of Virology, CAS. Pregnant mice with the same expected delivery date were acclimated in individually ventilated cages under standard conditions within an SPF environment. Food and water were available ad libitum. The mice were anaesthetized by isoflurane inhalation before any operation that may have caused potential discomfort. Before the experiment, suckling mice were randomly allocated into 6 distinct groups, namely, the healthy group, the vehicle group, the groups receiving DBM at 500 mg/kg (mpk) orally quaque die (QD), 200 mpk QD, and 100 mpk QD, and the group receiving VV116 at 25 mpk orally QD. Each group was comprised of 5–6 suckling mice. On the initiation day (Day 0) of the experiment, 5–6-day-old BALB/c suckling mice were intranasally infected with 1 × 10^4^ TCID50 of HCoV-OC43. Two hours postinoculation, the mice were orally treated with either a placebo or varying doses of the drugs on the basis of their respective groupings as outlined earlier. This oral treatment regimen was continued for several days. The body weight changes of the mice were monitored daily. On Day 5, all the mice in each group were sacrificed for virological and histopathological analyses. Various organs and tissues, such as the brain, spinal cord, lungs, and kidneys, were collected from the mice on ice. Portions of the organs and tissues were homogenized with DMEM and subsequently centrifuged at 3000 rpm for 10 min at 4 °C. Viral and host RNA from the organs and tissues were extracted and analysed as described above. For histologic examination, mouse organs and tissues were promptly collected after euthanasia and preserved in 4% paraformaldehyde for more than 5 days. The tissues were subsequently embedded in 3.5-mm paraffin. Fixed tissue samples were subjected to haematoxylin-eosin (H&E) and immunofluorescence staining to detect the HCoV-OC43 antigen with anti-HCoV-OC43 nucleocapsid protein rabbit serum (ABclonal). The images were captured with a Pannoramic MIDI system (3DHISTECH, Budapest) and FV1200 confocal microscope (Olympus). For the survival experiment, the mice were treated for 7 consecutive days after infection and euthanized on Day 14. Body weight changes were recorded daily. Owing to animal discomfort, some of the mice were euthanized before Day 14. Experiments involving authentic viruses were performed in an animal biosafety level 2 facility (ABSL-2) at the Wuhan Institute of Virology, CAS.

### Immunoplaque assay

RD cells were preseeded onto 24-well plates and incubated at 37 °C for 24 hours until they reached 90% confluence. The supernatants from the homogenized organs and tissues were serially diluted 10-fold in DMEM. Subsequently, 50 μL of each dilution was added to the wells. Following a 1-hour incubation at 37 °C, the inoculum was removed, and 200 μL of overlay medium (DMEM containing 2% FBS and 1.5% carboxymethyl cellulose) was added to the wells. Four days postinfection, the cells were fixed with 4% paraformaldehyde for 2 hours, followed by three washes with phosphate-buffered saline (PBS). The fixed cells were then permeabilized with 0.5% Triton X-100 for 30 minutes and blocked with 5% bovine serum albumin (BSA) for 30 minutes at room temperature. The cells were subsequently incubated with anti-HCoV-OC43 nucleocapsid protein rabbit serum (ABclonal, SA00001–2) as the primary antibody and HRP-conjugated anti-rabbit IgG (Proteintech) as the secondary antibody, followed by staining with a DAB staining kit (TIANGEN, PA110).

### Pharmacokinetic studies of DBM

The PK studies were conducted at Wuhan Hongren Biopharmaceutical, Inc. Eight SD rats were randomly divided into three groups and fasted for 12 h prior to dosing. The first group, consisting of three rats, received 10 mg/kg DBM via intravenous (IV) injection, whereas the second group, also consisting of three rats, received 200 mg/kg DBM orally (PO). Two rats served as the blank control. Blood samples were collected into EDTA-K2 tubes and centrifuged at 11,000 rpm for 10 min. The plasma was separated and stored at -70 °C for subsequent analysis. All procedures were conducted in an ice water bath. The concentration of analyte in the plasma was determined using liquid chromatography with tandem mass spectrometry (LC-MS/MS).

### Quantitative proteomics

The quantitative proteomics studies were conducted at Wuhan SpecAlly Life Technology Co. Ltd. Huh-7 cells were seeded into 6-well plates and incubated overnight at 37 °C. The culture medium was replaced with fresh culture medium containing either 20 μM DBM or DMSO as a control. After incubation for 48 hours, the culture medium was discarded, and the cells were washed three times with PBS. The cells were gently harvested into 1.5 ml microcentrifuge tubes and incubated at 60 °C for 1 hour with lysis buffer (1% SDC/100 mM Tris-HCl, pH = 8.5/10 mM TCEP/40 mM CAA) for protein reduction and alkylation. An equal volume of ddH_2_O was added to dilute the lysate, followed by overnight protein digestion with trypsin. The next day, TFA was added to terminate the digestion. After centrifugation, the supernatant was subjected to peptide purification with a custom-made SDB-RPS desalting column. The peptide eluate was vacuum-dried and stored at -20 °C for later use. All the samples were analysed via LC-MS/MS with an UltiMate 3000 RSLCnano (Thermo) coupled with a Q Exactive HF (Thermo).

### Western blot analysis

The cells were washed three times with PBS before being lysed on ice in RIPA lysis buffer (Beyotime, P0013C) containing PMSF (Acmec, AP0100). The lysates were mixed with SDS-PAGE sample loading buffer (Beyotime, P0015) and boiled for 10 minutes. The denatured proteins were separated by SDS-PAGE and then transferred onto a polyvinylidene difluoride (PVDF) membrane (Bio-Rad, 1620177). Next, the membrane was incubated with primary antibodies, followed by horseradish peroxidase (HRP)-conjugated secondary antibodies. The protein bands were visualized with Immobilon ECL UltraPlus Western HRP Substrate (Millipore, WBULP) and scanned with a ChemiDoc Imaging System (Bio-Rad).

### Time-of-addition assay

To evaluate the effects of DBM on the life cycle of HCoV-229E and HCoV-OC43, fresh culture medium containing 0.6 μM DBM, 0.1 μM remdesivir (RDV), or 10 μM chloroquine (CQ) was supplemented with preseeded cells at different time points relative to infection (entry group: 0 to –2 hours; postinfection group: 2–12 hours; and full-time group: 0 to –12 hours). DMSO served as the control. At 24 hours postinfection, the number of intracellular virus RNA copies was quantified by qRT-PCR as previously described.

### Viral binding assay

The cells were seeded onto 24-well plates and incubated overnight at 37 °C. Serially diluted DBM was added to the wells and incubated with the cells for 1 hour before infection at an MOI of 50. Following inoculation, the plates were placed on ice for two hours. The cells were washed three times with PBS before total RNA was extracted and analysed by qRT-PCR as previously described.

### Viral entry and internalization assays

The cells were seeded at a low density onto 35 mm glass bottom dishes with 10 mm microwells (Cellvis) and incubated overnight at 37 °C. One hour before infection, the culture medium was supplemented with DBM, and chloroquine (CQ) was added to prevent fusion. The cells were then inoculated with viruses at an MOI of 50, placed on ice for 1 hour, and subsequently incubated at 37 °C. The cells were washed three times with PBS buffer and fixed with 4% paraformaldehyde for 2 hours, followed by another three washes. The fixed cells were then permeabilized with 0.5% Triton X-100 for 30 minutes and blocked with 5% bovine serum albumin (BSA) for 30 minutes at room temperature. The cells were then incubated with an anti-HCoV-OC43 nucleocapsid antibody (Abcam, ab309964) as the primary antibody and 488-conjugated anti-human IgG (Proteintech, CL488–10284) as the secondary antibody. Images were obtained with the Dragonfly High-Speed Confocal Microscope Systems (Andor). The number of intracellular virus particles was quantified with ImageJ software.

### Cell-cell fusion assays

HEK293T cells were preseeded and cotransfected with plasmids encoding the HCoV-OC43 spike protein (including the ectodomain and transmembrane domain, ECD & TMD) and eGFP. At 24 hours posttransfection, the HEK293T cells were suspended, mixed with RD cells, and seeded onto 24-well plates. The mixed cells were cultured at 37 °C for 12 hours and washed three times with PBS before being treated with FBS-free DMEM containing TPCK-treated trypsin (Sigma-Aldrich, 100 ng/mL) for 1 h. Subsequently, the cells were incubated with fresh culture medium for 16 hours before imaging.

### Transient transfections and RNA interference

Plasmid transfection was performed with Lipofectamine 3000 (Invitrogen) according to the manufacturer’s instructions. For RNA interference, cells were transfected with siRNAs with Lipofectamine RNAiMAX following the manufacturer’s guidelines. Scramble siRNA was used as the control. The siRNAs used in this research were as follows: IDH3G-1, F: 5’- GCAAGAGUAUCGCCAAUAATT -3’ and R: 5’- UUAUUGGCGAUACUCUUGCTT -3’; IDH3G-2, F: 5’- GCACGUGAGUUCCAAUGCUTT -3’ and R: 5’- AGCAUUGGAACUCACGUGCTT -3’; CYP1A1-1, F: 5’- GAUCCCAUCUGCCCUAUAUTT -3’ and R: 5’- AUAUAGGGCAGAUGGGAUCTT -3’; CYP1A1-2, F: 5’- GGGUUUGACACAGUCACAATT -3’ and R: 5’- UUGUGACUGUGUCAAACCCTT -3’; ELAC2–1, F: 5’- GCUUCCAAAGUGUGUACUUTT -3’ and R: 5’- AAGUACACACUUUGGAAGCTT -3’; ELAC2–2, F: 5’- GCAGAGGGAUGCCAUUAUUTT -3’ and R: 5’- AAUAAUGGCAUCCCUCUGCTT -3’; UBP1–1, F: 5’- GCACCCACCCUUUCAGUAUTT -3’ and R: 5’- AUACUGAAAGGGUGGGUGCTT -3’; UBP1–2, F: 5’- GCAUCAUAAGGGUUGUAUUTT -3’ and R: 5’- AAUACAACCCUUAUGAUGCTT -3’; ALG11–1, F: 5’- GCUCUCUGUAGUGAAGAAUTT -3’ and R: 5’- AUUCUUCACUACAGAGAGCTT -3’; ALG11–2, F: 5’- GGUGCCUGUGCAAGUUGUUTT -3’ and R: 5’- AACAACUUGCACAGGCACCTT -3’; CASP3–1, F: 5’- CCGACAAGCUUGAAUUUAUTT -3’ and R: 5’- AUAAAUUCAAGCUUGUCGGTT -3’; CASP3–2, F: 5’- CCCUGGACAACAGUUAUAATT -3’ and R: 5’- UUAUAACUGUUGUCCAGGGTT -3’; CASP7–1, F: 5’- GUGCCAAGAUGCAAGAUCUTT -3’ and R: 5’- AGAUCUUGCAUCUUGGCACTT -3’; CASP7–2, F: 5’- GAGGAUUCAGCAAAUGAAGTT -3’ and R: 5’- CUUCAUUUGCUGAAUCCUCTT -3’; CASP6–1, F: 5’- CUCUGUUGCAGAAGGAUAUTT -3’ and R: 5’- AUAUCCUUCUGCAACAGAGTT -3’; CASP6–2, F: 5’- CUGCGCAGAUAGAGACAAUTT -3’ and R: 5’- AUUGUCUCUAUCUGCGCAGTT -3’; and CASP6–3, F: 5’- GGCAAUCACAUUUAUGCAUTT -3’ and R: 5’- AUGCAUAAAUGUGAUUGCCTT -3’.

### Caspase-6 activity assay

Caspase-6 enzyme activity was measured with the Caspase-6 Activity Assay Kit (Beyotime, C1135) following the manufacturer’s protocol. The cells pretreated with DMSO, DBM, or Z-VEID-FMK were digested with trypsin and washed three times with PBS before being collected by centrifugation. The cell samples were lysed on ice for 15 minutes using the lysis solution provided in the kit. The lysates were subsequently centrifuged at 16,000–20,000 × g for 10–15 minutes at 4 °C, and the supernatant was collected for the enzyme activity assay.

### CRISPR/Cas9-mediated knockout (KO) *in vitro*

Double-stranded guide RNA (gRNA) sequences were designed using an online tool (http://crispr.mit.edu). *CASP6*-targeting oligonucleotide pairs (forward sequence: 5’ -CACCGAAAAGTACAAAATGGACCAC -3’ and reverse sequence: 5’ -AAACGTGGTCCATTTTGTACTTTTC -3’) were synthesised and cloned into LentiCRISPR v2 plasmids. Scrambled control LentiCRISPR v2 plasmids were generated using the same approach. For lentiviral packaging, HEK293T cells were seeded in six-well plates and transfected with either *CASP6*-targeting LentiCRISPR v2 plasmids or scrambled control LentiCRISPR v2 plasmids, along with two other packaging plasmids pMD2.G and pCMV-dR8.91. Six hours post-transfection, the culture medium was replaced with maintenance medium. Following a further 42 hours of incubation, the supernatant containing the recombinant lentivirus was harvested and filtered through a sterile 0.45 μm hydrophilic PVDF membrane syringe filter (Millipore, SLHV033R). RD cells were infected with lentivirus-containing supernatant. 96 hours post-infection, cells were subjected to selection with 2 μg/ml puromycin for 7 days. Successful knockout of caspase-6 was confirmed by Western blot analysis.

## Supporting information

S1 Fig EC_50_ and CC_50_ evaluation of natural compounds. **A**) Half-maximal effective concentration (EC_50_) values for HCoV-229E and HCoV-OC43, and 50% cytotoxic concentration (CC_50_) values of the natural compounds in Huh-7 and RD cells. **B**) EC_50_ values of DBM in inhibiting HCoV-NL63. **C**) Cytotoxicity data of the five natural products in MRC-5, HEK293T-AT, and Vero E6 cell lines. **D**) Table of CC_50_ values for the five natural products.
(TIF)

S2 Fig Pharmacokinetic and metabolic stability study of DBM. **A**) Pharmacokinetic parameters of DBM following oral administration in rats. **B**) Metabolic stability data of DBM in human and mouse liver microsomes. The experiments were repeated three times independently with similar results.
(TIF)

S3 Fig DBM reduces the quantity of HCoV-OC43 nucleocapsid proteins in mouse. **A**) Immunofluorescence staining of spinal cord and kidney sections from mice treated with different regimens. HCoV-OC43 nucleocapsid proteins were detected by immunofluorescence (green). Cell nuclei were stained with DAPI (blue).
(TIF)

S4 Fig The efficacy of DBM against organs and tissues damage. **A**) Cytokine gene expression measured in the brains and spinal cords at 5 dpi. The relative expression levels of *CCL2*, *CXCL10*, *IL-1β*, *IL-6*, and *TNF-α* were compared to those in healthy mice. **B**) H&E-stained sections of the brains and lungs of mice following 5 days of treatment. The accumulation of immune effector cells (black arrows) and severe lesions (red arrows) in the brain, along with pulmonary fibrosis (black arrows), pulmonary oedema (red arrows), and the formation of sputum (green arrows) in the lungs, were observed. The experiments were repeated three times independently with similar results.
(TIF)

S5 Fig DBM treatment does not impair viral entry and budding. **A**) Efficacy of DBM, remdesivir (RDV), and chloroquine (CQ) in inhibiting virus replication at different stages of administration. **B**) Capacity of DBM in inhibiting virion binding to the cell surface. **C**) Fluorescence images of cell-cell fusion induced by the transient expression of HCoV-OC43 spike proteins and eGFP (green) in RD cells. The scale bar represents 50 μM. **D**) Confocal fluorescence microscopy images showing HCoV-OC43 internalization. **E**) Quantification of intracellular viral particles per cell, based on images obtained from confocal fluorescence microscopy. **F**) Effect of DBM treatment on the proportion of release of HCoV-OC43 RNA. Data were statistically analysed with Student’s T test. *: p ≤ 0.05, **: p ≤ 0.01, ***: p ≤ 0.001, ****: p ≤ 0.0001; ns, not significant. The experiments were repeated three times independently with similar results.
(TIF)

S6 Fig Caspase-3 and caspase-4 does not inhibit OCoV-OC43 replication. **A**) Relative mRNA content following siRNA knockdown. **B**) Antiviral efficacy of knockdown of *CASP3*, *CASP6* or *CASP7*. **C**) EC_50_ values of caspase-3/7 inhibitor I in inhibiting HCoV-OC43.
(TIF)

S7 Fig Screening for drug-resistant strains of HCoV-OC43. **A**) Ratio of DBM concentration used during successive HCoV-OC43 passages to the EC_50_ value. **B**) EC_50_ values of DBM against HCoV-OC43 strains that were passed for 17 generations in the presence of DBM or DMSO. The experiments were repeated three times independently with similar results.
(TIF)

S8 Fig DBM alleviated the suppression of IFN signalling induced by coronaviruses nucleocapsid proteins and caspase-6. **A)** *IFNB1* expression in SARS-CoV-2 variant BA.5-infected/non-infected 293T-AT and VeroE6 cells. **B**) Relative expression of representative IFN-stimulated genes *IFIT1*, *IFITM3*, *OAS1*, and *TRIM22* analysed by qPCR. **C**) Relative expression of representative IFN-stimulated genes *IFIT1*, *IFITM3*, *OAS1*, and *TRIM22* analysed by qPCR. **D**) *IFNB1* expression in HCoV-OC43-infected/non-infected cells. **E**) ISGs expression in HCoV-OC43-infected/non-infected cells. The experiments were repeated three times independently with similar results.
(TIF)

S9 Fig Model diagram of the mechanism of inhibition of coronavirus replication by DBM. **A)** The coronavirus genome is released into the host cell and subsequently hijacks the host protein translation machinery to synthesise structural proteins such as coronavirus N protein, which is cleaved by caspase-6 to form protein fragments that can inhibit IRF-3-mediated downstream type I interferon gene expression and signal transduction. After entering the host cells, DBM can reduce the production of N protein cleavage products by down-regulating the load of caspase-6 protein at the mRNA level, which antagonises the natural immunosuppression dominated by coronavirus N protein and caspase-6. The schematic was created by the authors using Adobe Illustrator 2022.
(TIF)

S1 Data Raw data. All data generated and analysed in this study.
(XLSX)
