# Supplementary figures and images for: Discovery and mechanistic insights of dibenzoylmethane as a broad spectrum inhibitor of coronavirus
Source: PLoS Pathog. 2025 Sep 8;21(9):e1013492. doi: 10.1371/journal.ppat.1013492 (PMC12431657; doi:10.1371/journal.ppat.1013492)

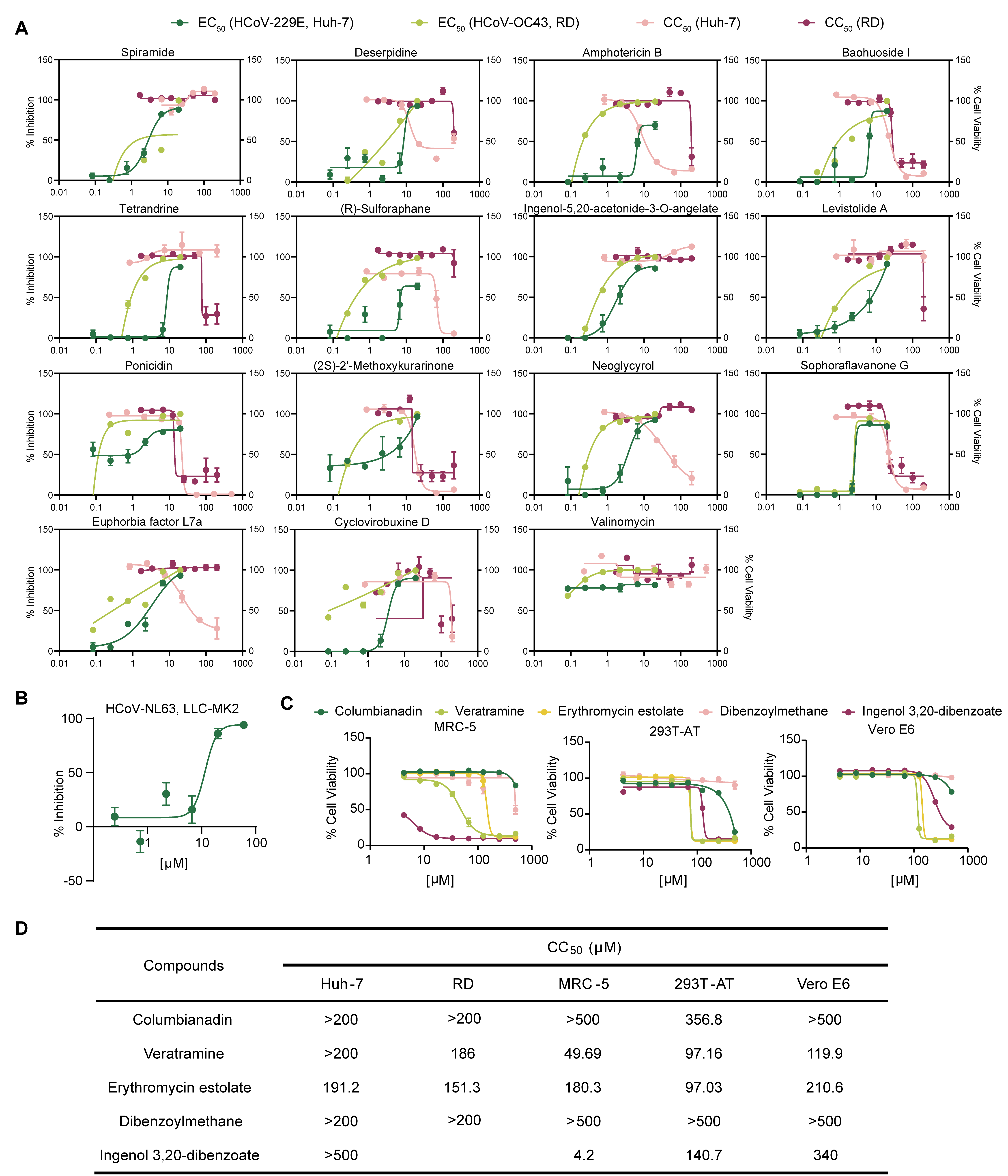

Supplement: S1 Fig — A) Half-maximal effective concentration (EC50) values for HCoV-229E and HCoV-OC43, and 50% cytotoxic concentration (CC50) values of the natural compounds in Huh-7 and RD cells. B) EC50 values of DBM in inhibiting HCoV-NL63. C) Cytotoxicity data of the five natural products in MRC-5, HEK293T-AT, and Vero E6 cell lines. D) Table of CC50 values for the five natural products. (TIF) [file ppat.1013492.s001.tif]

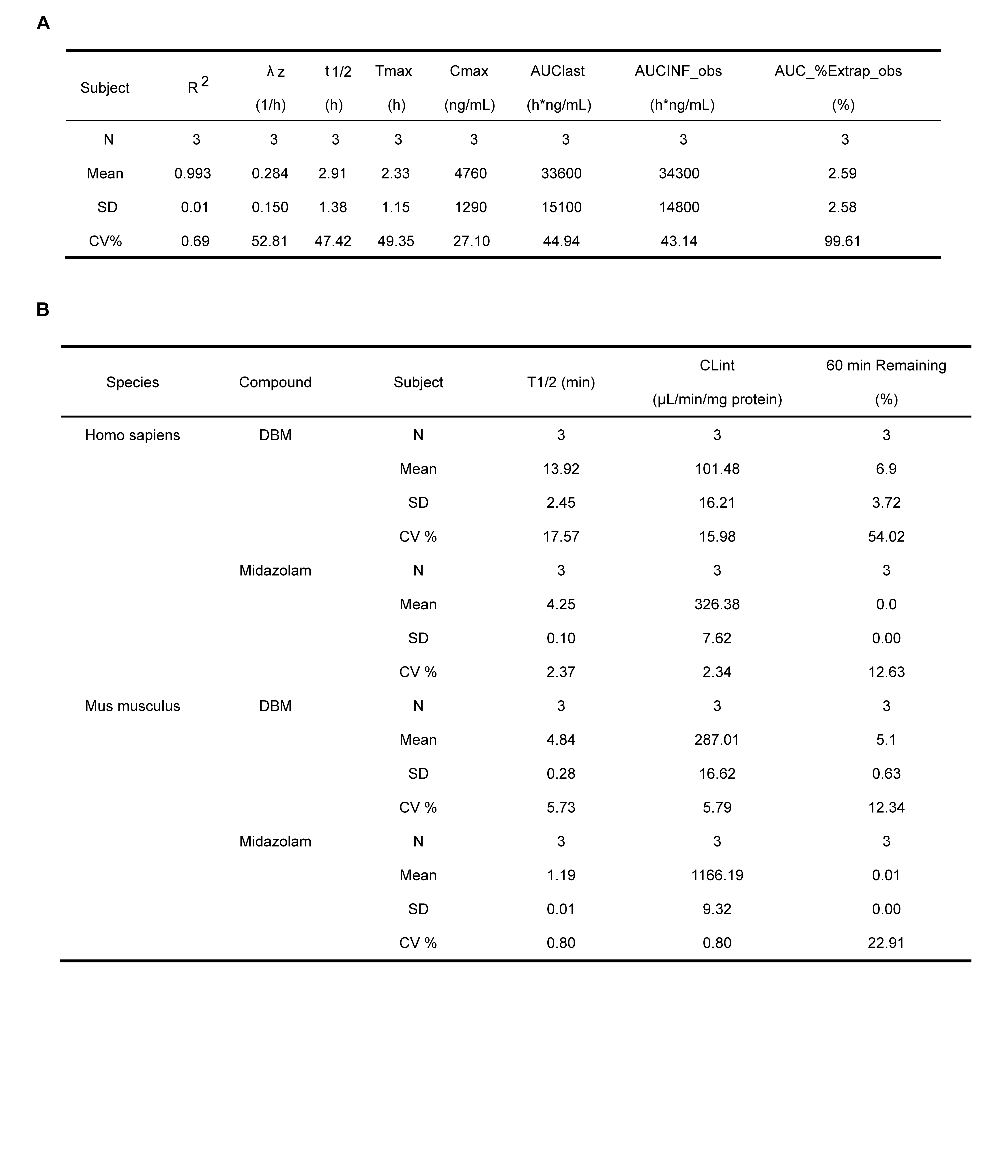

Supplement: S2 Fig — A) Pharmacokinetic parameters of DBM following oral administration in rats. B) Metabolic stability data of DBM in human and mouse liver microsomes. The experiments were repeated three times independently with similar results. (TIF) [file ppat.1013492.s002.tif]

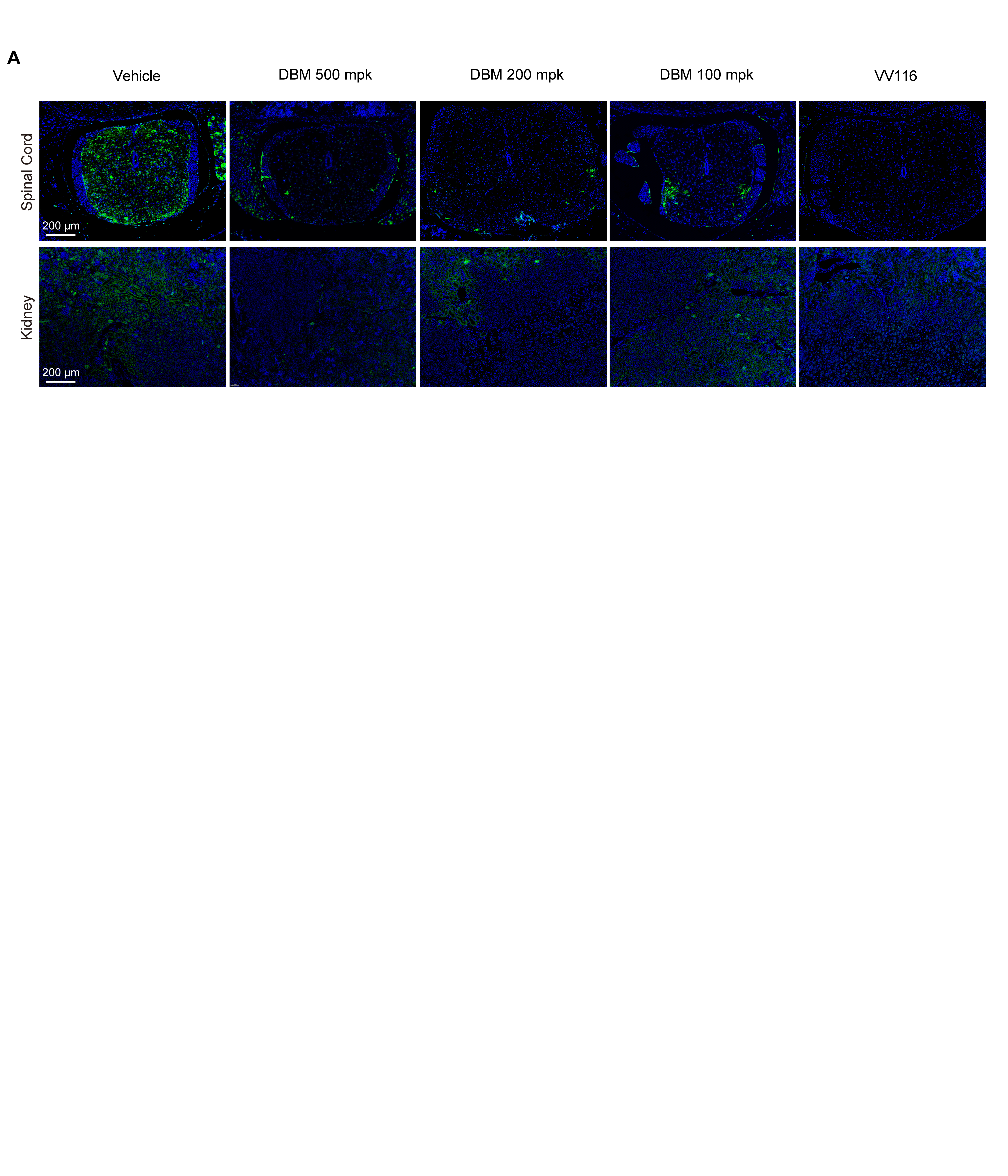

Supplement: S3 Fig — A) Immunofluorescence staining of spinal cord and kidney sections from mice treated with different regimens. HCoV-OC43 nucleocapsid proteins were detected by immunofluorescence (green). Cell nuclei were stained with DAPI (blue). (TIF) [file ppat.1013492.s003.tif]

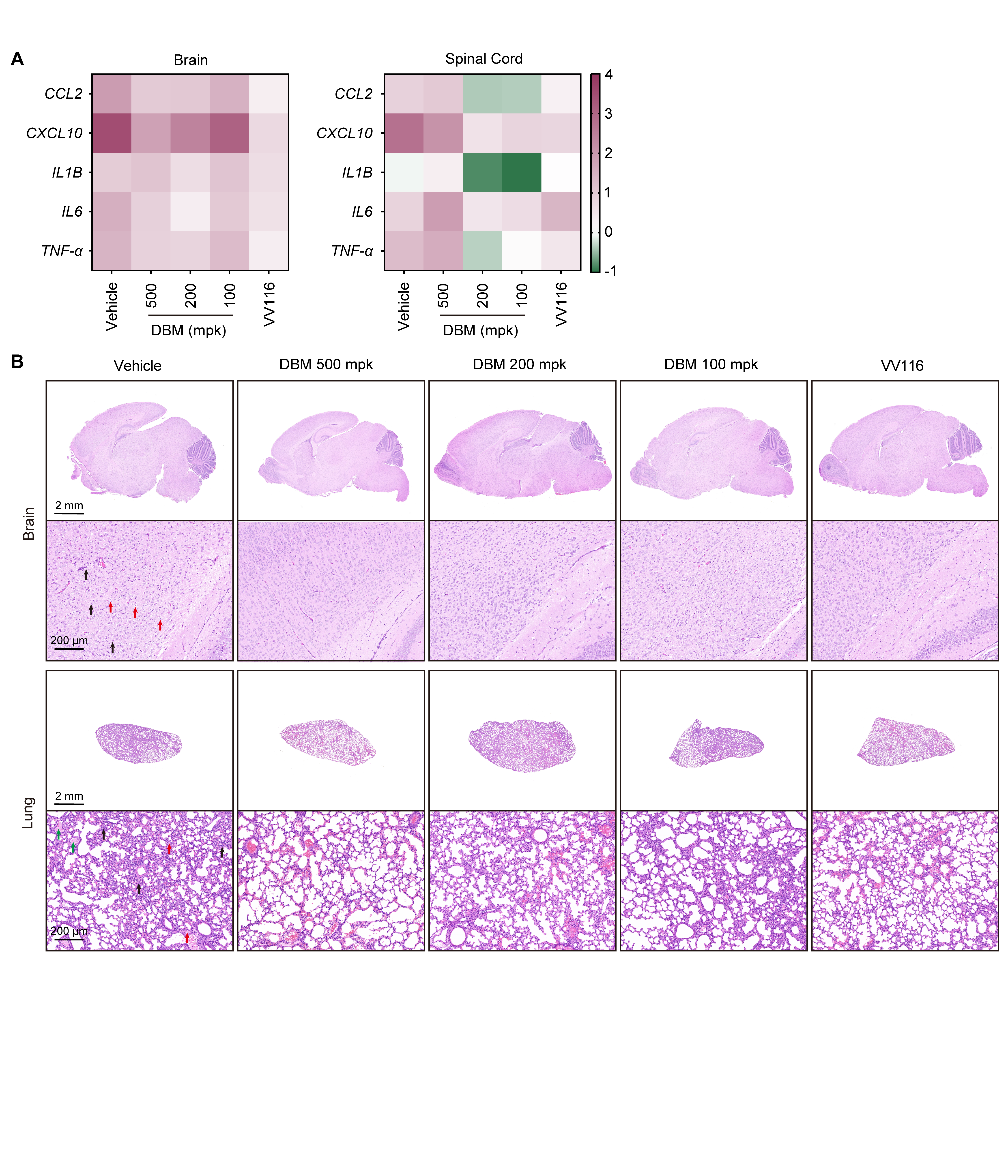

Supplement: S4 Fig — A) Cytokine gene expression measured in the brains and spinal cords at 5 dpi. The relative expression levels of CCL2, CXCL10, IL-1β, IL-6, and TNF-α were compared to those in healthy mice. B) H&E-stained sections of the brains and lungs of mice following 5 days of treatment. The accumulation of immune effector cells (black arrows) and severe lesions (red arrows) in the brain, along with pulmonary fibrosis (black arrows), pulmonary oedema (red arrows), and the formation of sputum (green arrows) in the lungs, were observed. The experiments were repeated three times independently with similar results. (TIF) [file ppat.1013492.s004.tif]

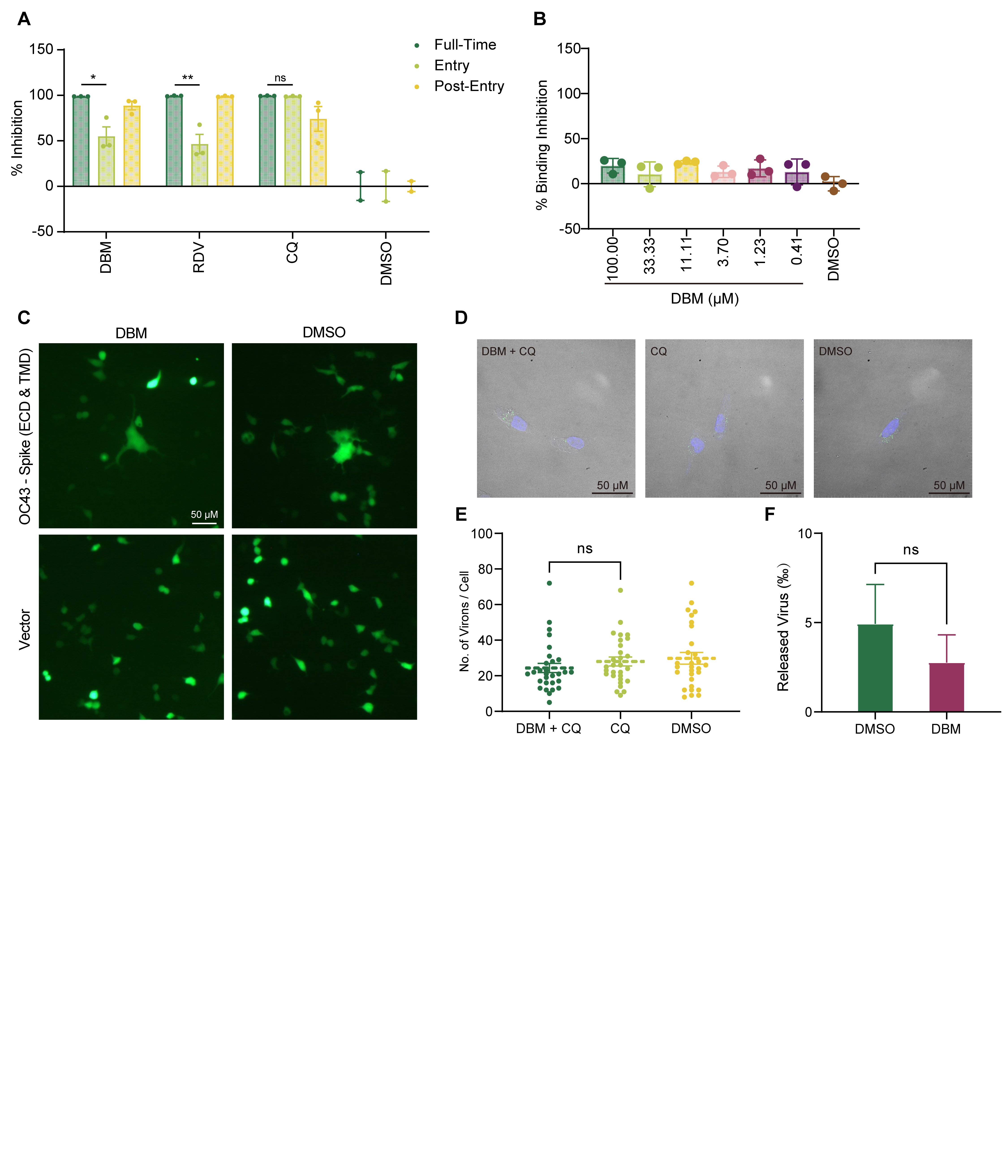

Supplement: S5 Fig — A) Efficacy of DBM, remdesivir (RDV), and chloroquine (CQ) in inhibiting virus replication at different stages of administration. B) Capacity of DBM in inhibiting virion binding to the cell surface. C) Fluorescence images of cell-cell fusion induced by the transient expression of HCoV-OC43 spike proteins and eGFP (green) in RD cells. The scale bar represents 50 μM. D) Confocal fluorescence microscopy images showing HCoV-OC43 internalization. E) Quantification of intracellular viral particles per cell, based on images obtained from confocal fluorescence microscopy. F) Effect of DBM treatment on the proportion of release of HCoV-OC43 RNA. Data were statistically analysed with Student’s T test. *: p ≤ 0.05, **: p ≤ 0.01, ***: p ≤ 0.001, ****: p ≤ 0.0001; ns, not significant. The experiments were repeated three times independently with similar results. (TIF) [file ppat.1013492.s005.tif]

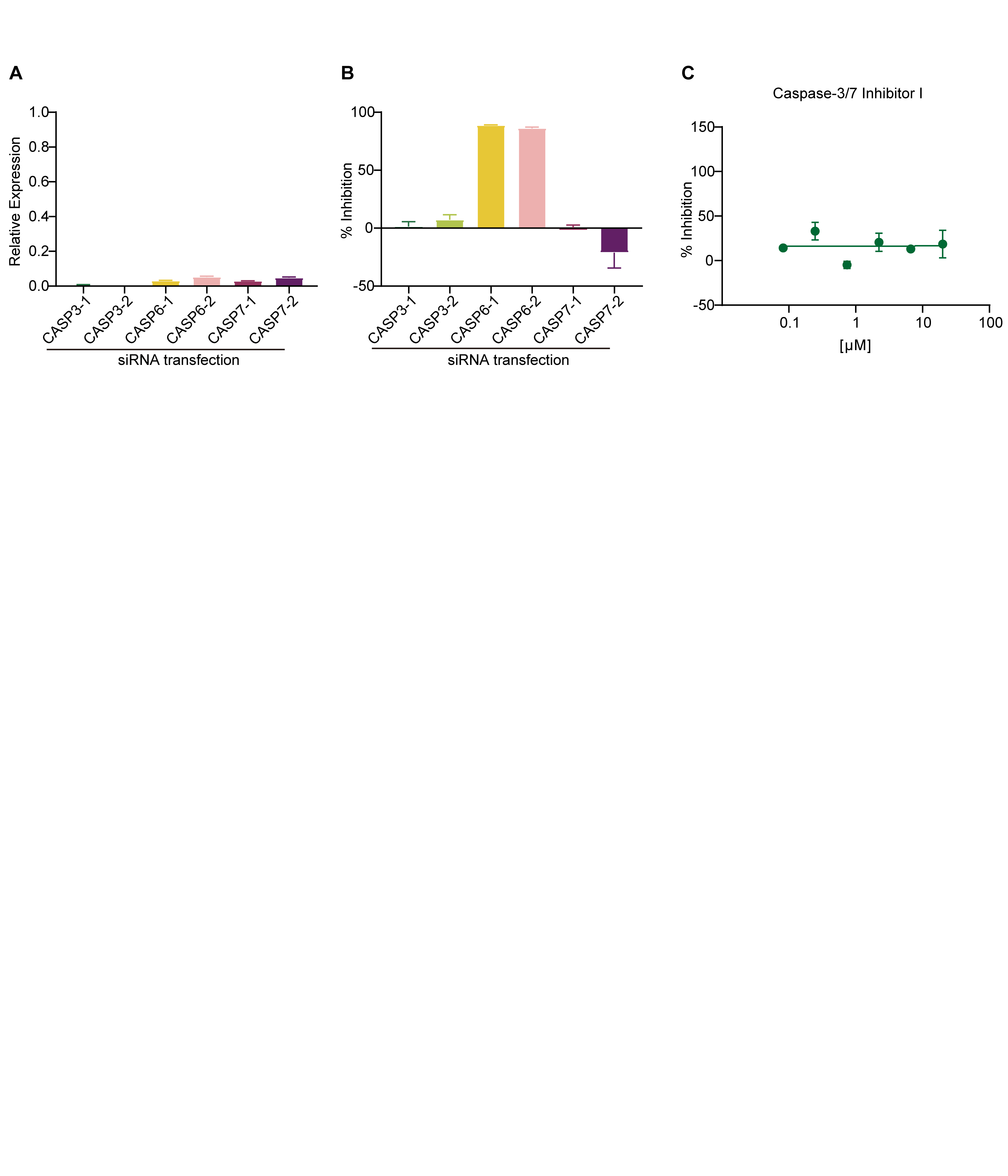

Supplement: S6 Fig — A) Relative mRNA content following siRNA knockdown. B) Antiviral efficacy of knockdown of CASP3, CASP6 or CASP7. C) EC50 values of caspase-3/7 inhibitor I in inhibiting HCoV-OC43. (TIF) [file ppat.1013492.s006.tif]

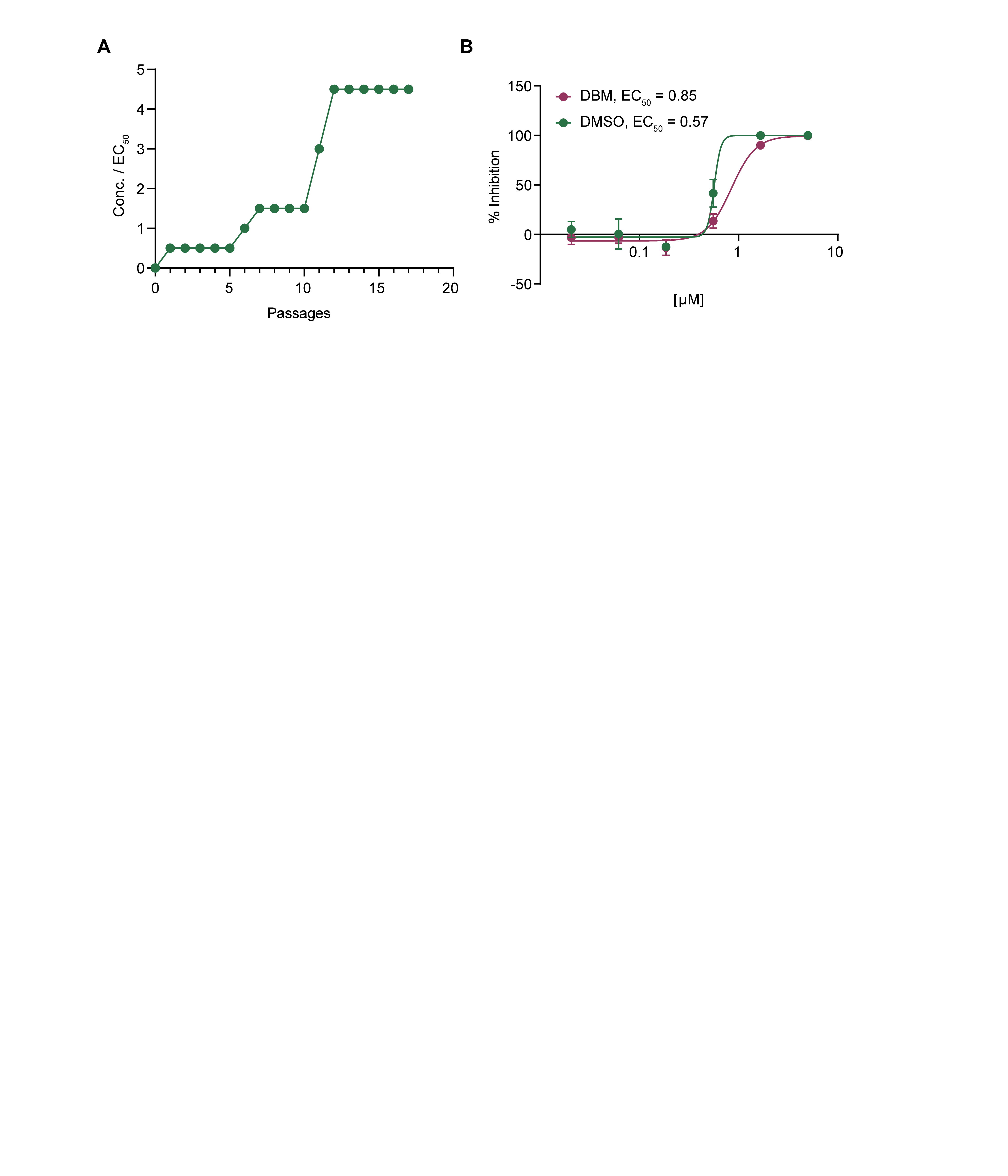

Supplement: S7 Fig — A) Ratio of DBM concentration used during successive HCoV-OC43 passages to the EC50 value. B) EC50 values of DBM against HCoV-OC43 strains that were passed for 17 generations in the presence of DBM or DMSO. The experiments were repeated three times independently with similar results. (TIF) [file ppat.1013492.s007.tif]

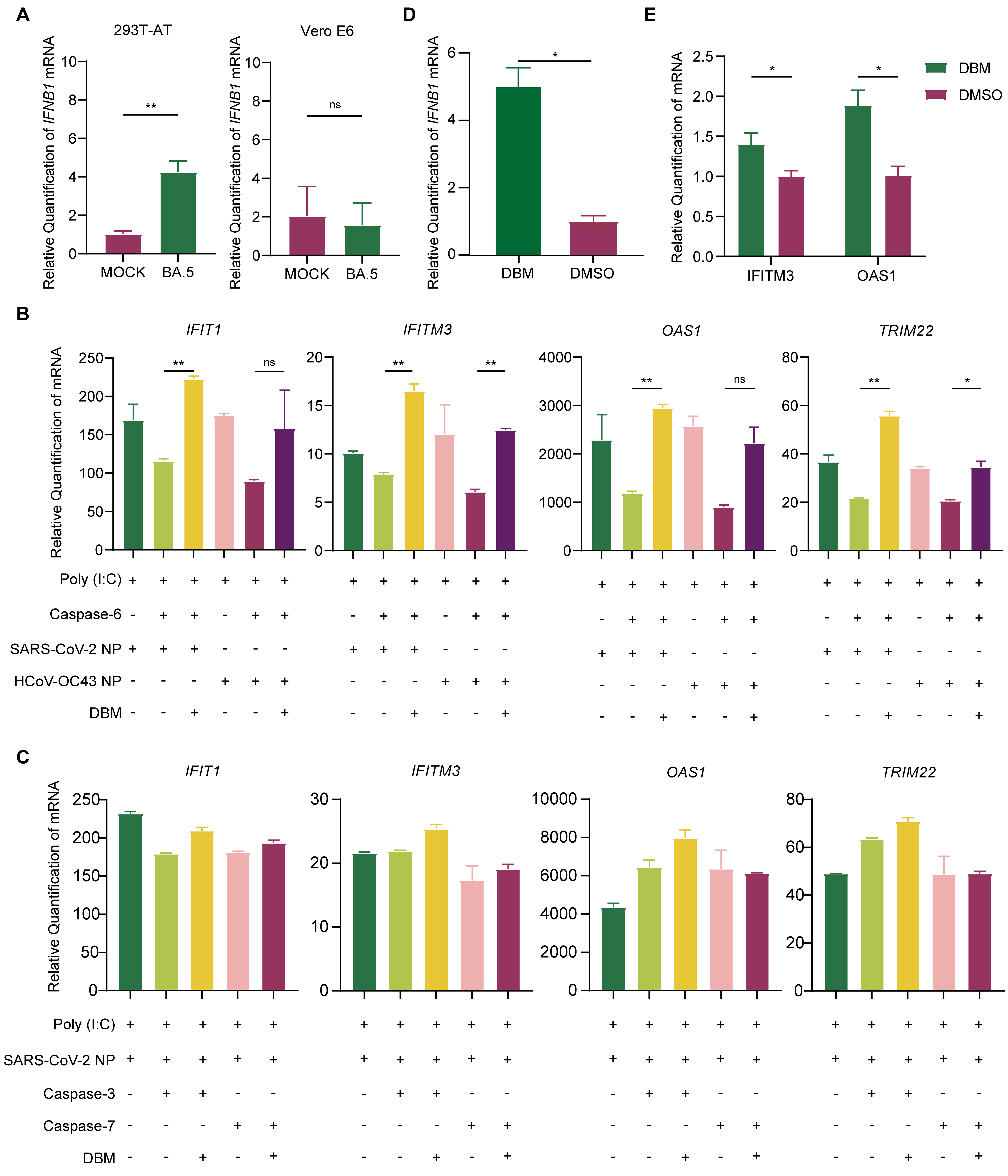

Supplement: S8 Fig — A) IFNB1 expression in SARS-CoV-2 variant BA.5-infected/non-infected 293T-AT and VeroE6 cells. B) Relative expression of representative IFN-stimulated genes IFIT1, IFITM3, OAS1, and TRIM22 analysed by qPCR. C) Relative expression of representative IFN-stimulated genes IFIT1, IFITM3, OAS1, and TRIM22 analysed by qPCR. D) IFNB1 expression in HCoV-OC43-infected/non-infected cells. E) ISGs expression in HCoV-OC43-infected/non-infected cells. The experiments were repeated three times independently with similar results. (TIF) [file ppat.1013492.s008.tif]

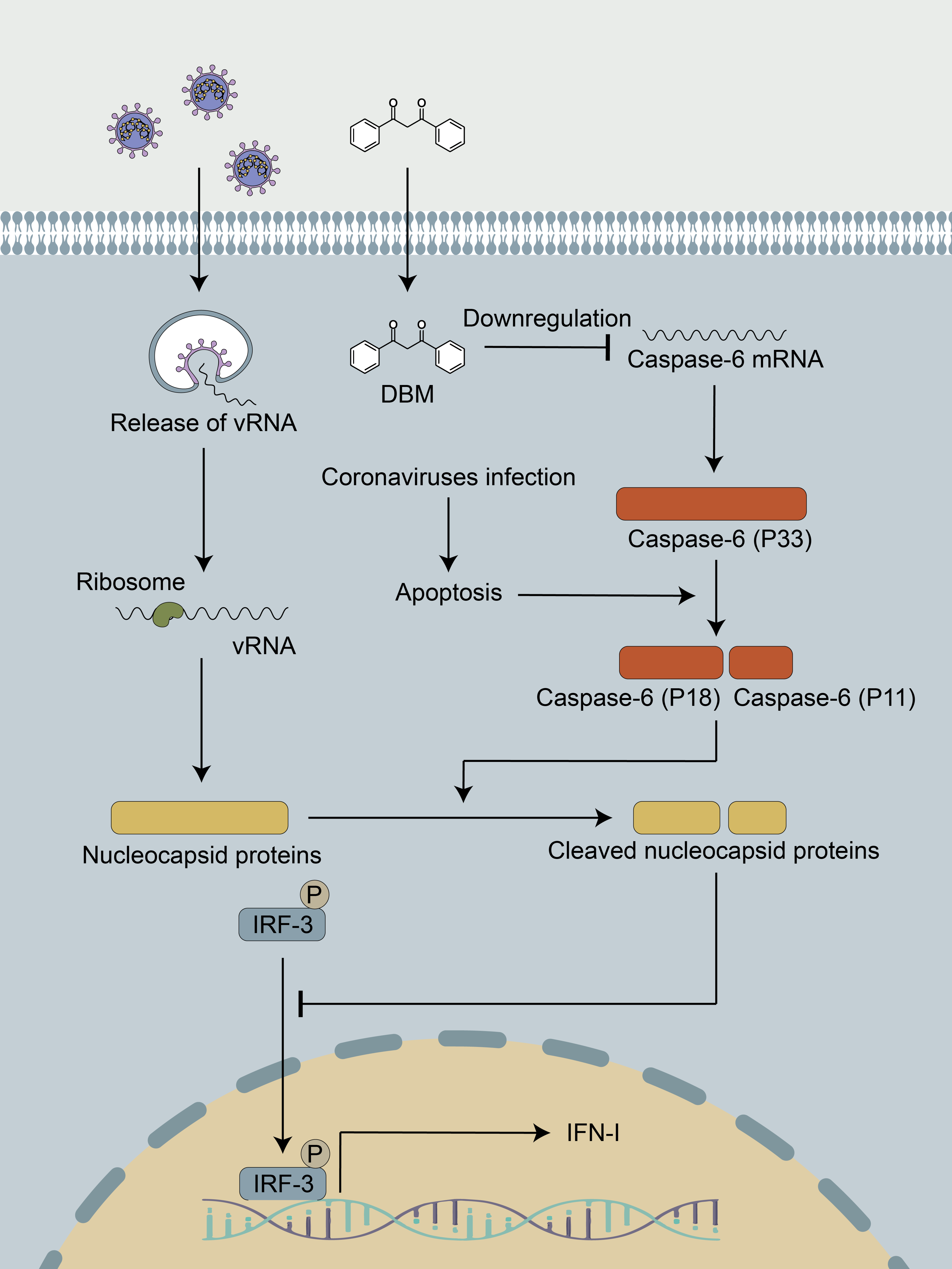

Supplement: S9 Fig — A) The coronavirus genome is released into the host cell and subsequently hijacks the host protein translation machinery to synthesise structural proteins such as coronavirus N protein, which is cleaved by caspase-6 to form protein fragments that can inhibit IRF-3-mediated downstream type I interferon gene expression and signal transduction. After entering the host cells, DBM can reduce the production of N protein cleavage products by down-regulating the load of caspase-6 protein at the mRNA level, which antagonises the natural immunosuppression dominated by coronavirus N protein and caspase-6. The schematic was created by the authors using Adobe Illustrator 2022. (TIF) [file ppat.1013492.s009.tif]
